# Ultrasonic Reporter of Kinase Activity

**DOI:** 10.1021/acsnano.6c14484

**Published:** 2026-09-09

**Authors:** Jee Won Yang, Zhiyang Jin, Ting-Yu Wang, Mikhail G. Shapiro

**Affiliations:** † Division of Chemistry and Chemical Engineering, 166586 California Institute of Technology, Pasadena, California 91125, United States; ‡ Division of Engineering and Applied Sciences, 6469 California Institute of Technology, Pasadena, California 91125, United States; § Proteome Exploration Laboratory, 6469 California Institute of Technology, Pasadena, California 91125, United States; ∥ Andrew and Peggy Cherng Department of Medical Engineering, California Institute of Technology, Pasadena, California 91125, United States; ⊥ Howard Hughes Medical Institute, Pasadena, California 91125, United States

**Keywords:** gas vesicles, molecular ultrasound imaging, genetically encoded biosensors, protein engineering, kinase activity

## Abstract

Protein kinases are an essential class of enzymes that regulate cellular signaling pathways, with their dysregulation implicated in pathologies such as cancer and neurodegenerative diseases. Despite the existence of high-performance fluorescent biosensors of kinase activity, it remains challenging to study the function and regulatory pathways of kinases in opaque tissues due to the limited tissue penetration of light. To address this limitation, we report an ultrasonic reporter of kinase activity (UReKA) designed to detect protein kinase A (PKA) activity by altering the ultrasound scattering of gas vesicles (GVs), a unique class of air-filled protein nanostructures. We engineer a GV shell protein to respond to PKA, demonstrate the functionality of UReKA both in purified protein format and expressed in mammalian cells, and showcase its capacity to monitor PKA signaling in response to pharmacological stimulation or genetic mutations. This approach provides a means to visualize cellular functional activity in opaque media, with broad potential for future applications in cancer biology, cellular development, and drug discovery.

## Graphical abstract

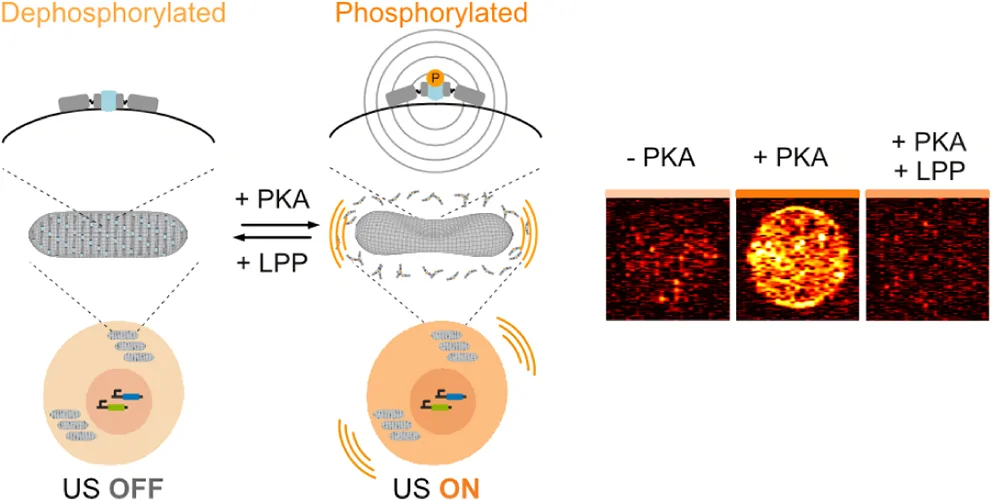

Kinases are a diverse family of enzymes that regulate a wide range of biological processes across virtually all cell types through phosphorylation,^1–3^ including signal transduction,^4^ metabolic homeostasis,^5^ immune regulation,^5^ and neuroprotection.^6^ Moreover, aberrant kinase signaling is implicated in numerous pathologies, such as cancer, chronic inflammation, diabetes, and neurological disorders,^6–8^ making this class of enzymes prime targets for therapeutic intervention.^8,9^ Hence, the ability to visualize kinase activity with high spatiotemporal precision is increasingly needed to study the dynamics of cellular signal transduction and to facilitate drug development and therapeutic monitoring.

Due to the central role of kinases in cellular function and dynamics, many imaging and analysis methods have been developed to understand their activity, substrate specificity, and function. However, traditional protein kinase assays, including phosphoproteomics^10^ and radiolabeled [^32^P] techniques,^11^ require cell lysis, preventing real-time observation of dynamic phosphorylation events. More recently, genetically encoded biosensors based on fluorescence, luminescence, Förster resonance energy transfer,^12^ and fluorescence lifetime imaging microscopy techniques have greatly improved spatiotemporal resolution in live cells^13,14^ and animal models.^15–17^ Yet, these optical techniques are inherently restricted by light scattering in tissues,^18^ limiting their applicability to study kinase activity in opaque samples. While the advent of a noninvasive kinase reporter based on magnetic resonance imaging^19,20^ provided an alternative modality with greater tissue penetration, the system was not expressed in cells or demonstrated intracellular enzyme activity sensing.

In contrast, ultrasound offers unique advantages for imaging cellular responses due to its ability to penetrate centimeters into opaque tissue while maintaining high spatial (∼100 μm) and temporal (∼1 ms) resolution.^21,22^ Genetically encoded ultrasound contrast agents have been developed from gas vesicles (GVs), air-filled protein nanostructures derived from buoyant microbes.^23^ These agents function as acoustic reporter genes^24–26^ and biosensors of protease activity^27^ and intracellular calcium,^28^ with their acoustic properties regulated by gas vesicle protein C (GvpC), an α-helical surface protein that stiffens the GV shell and controls buckling, collapse pressure, and nonlinear contrast generation.^29–33^ In this study, we report an ultrasonic reporter of kinase activity (UReKA) engineered to enable dynamic, noninvasive monitoring of intracellular protein kinase A (PKA) activity.

## Results and Discussion

### UReKA Design and Identification

To demonstrate the feasibility of UReKA, we selected PKA as a model sensing target due to its involvement in diverse cellular pathways.^34–37^ We hypothesized that phosphorylation-induced charge and conformational changes within a surface-binding protein could modulate the mechanical properties of GVs, leading to a change in nonlinear ultrasound contrast (Figure 1a). To test this concept, we engineered variants of GvpC, a structural protein known to mechanically reinforce the GV shell, to incorporate various PKA substrate motifs.^13,38^ We hypothesized that phosphorylation of the embedded serine/threonine residues would reduce GvpC’s affinity for the GV surface, resulting in a more deformable shell and a corresponding enhancement in nonlinear acoustic response.^30^ Conversely, we envisioned the activity of a phosphatase reversing phosphorylation, returning GvpC to the GV shell, and enabling reversible imaging of phosphorylation dynamics.

**Figure 1:**
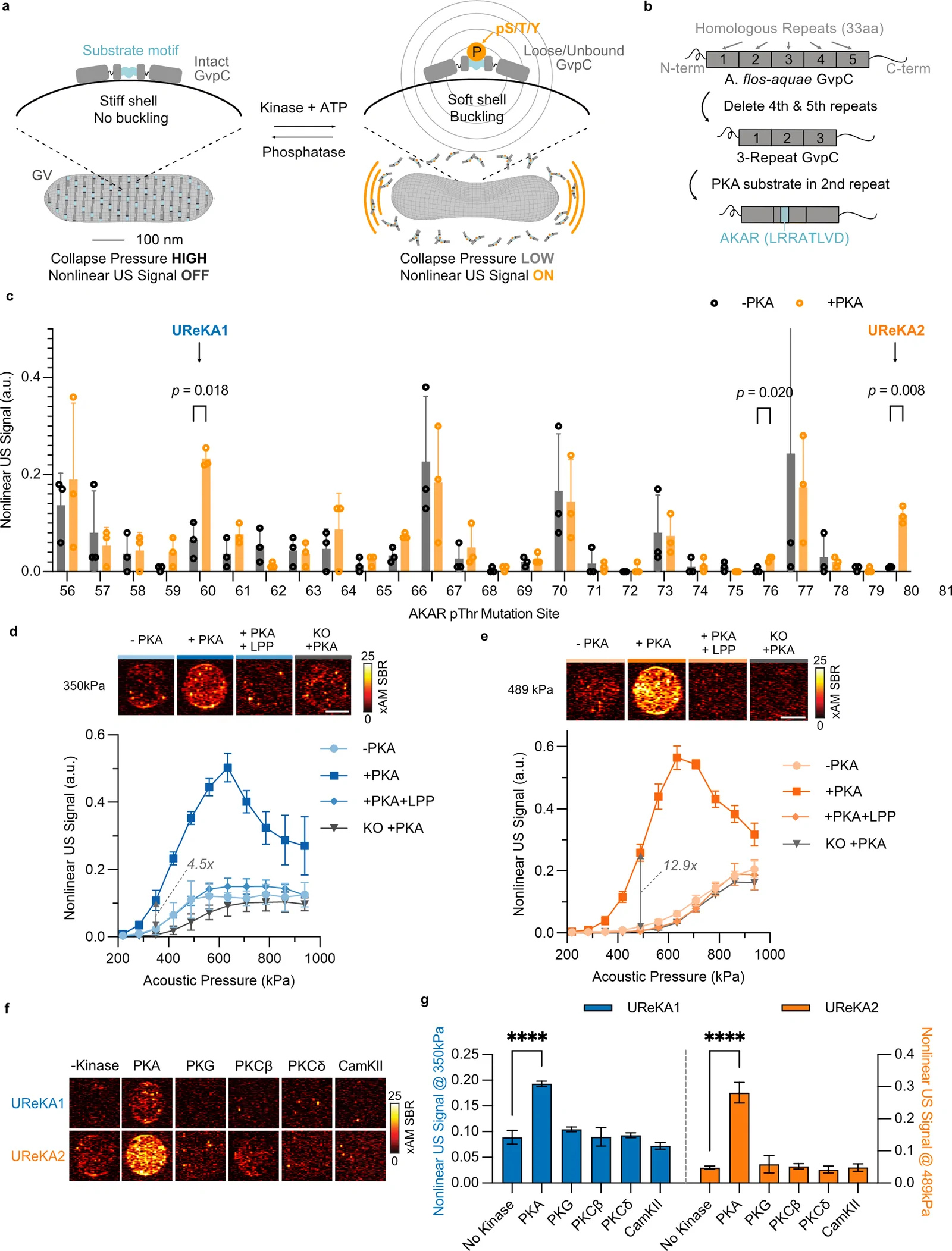
Identification and characterization of UReKA. (a) General design schematics of UReKA and its proposed mechanism of action. (b) Schematics of the UReKA variant GvpC design process. (c) Nonlinear ultrasound normalized by B-mode signal screening of AKAR-UReKA variants at various phosphorylated threonine (pThr) positions (56–81) in 3R GvpC at 419 kPa. Statistical significance was assessed for each mutant by a two-tailed paired *t* test comparing matched biological replicates for −PKA and + PKA conditions; *p*-values are unadjusted and are provided as exploratory screening statistics (*n* = 3). (d) (Top) Representative ultrasound images of an agarose phantom containing UReKA1 and nonlinear ultrasound signal at 350 kPa. Scale bar = 1 mm. (Bottom) Normalized nonlinear ultrasound signal as a function of acoustic pressure for UReKA1, without PKA, with PKA, with PKA and LPP, and with KO control (*n* = 3). (e) (Top) Representative xAM ultrasound images of an agarose phantom containing UReKA2 and nonlinear ultrasound signal at 489 kPa. Scale bar = 1 mm. (Bottom) Normalized nonlinear ultrasound signal as a function of acoustic pressure for UReKA2, without PKA, with PKA, with PKA and LPP, and with KO control (*n* = 3). (f) Representative ultrasound images of agarose phantom containing UReKA1 and UReKA2 at 350 and 489 kPa, respectively, incubated with PKA, PKG, PKCβ, PKCδ, and CaMKII. Scale bar = 1 mm. (g) Normalized nonlinear ultrasound signal for UReKA1 and UReKA2 at 350 and 489 kPa, respectively, with PKA, PKG, PKCβ, PKCδ, and CaMKII (*n* = 3).

Based on previous experience in GvpC engineering,^26–28^ we used a three-repeat GvpC (3R-GvpC) construct as the backbone, in which the last two of the five 33-amino-acid homologous regions in the wild-type *Anabaena flos-aquae* (Ana) GvpC gene were deleted (Figure 1b). A library of GvpC variants was generated by inserting the A kinase ctivity reporter (AKAR) PKA substrate sequence (LRRATLVD)^13^ into individual positions within the second repeat (residues 56–81), replacing the wild-type amino acids at the corresponding positions. Screening of these constructs revealed position-dependent changes in nonlinear ultrasound intensity following incubation in the absence or presence of PKA (Figure 1c). In this initial screen, two variants containing the engineered threonine at positions 60 and 81 exhibited the largest phosphorylation-dependent signal enhancement under cross-propagating amplitude modulation (xAM)^30^ nonlinear imaging at 419 kPa peak positive transmit pressure and were then selected for subsequent detailed characterization as UReKA1 and UReKA2, respectively. Notably, several additional variants were phosphorylated under the assay conditions but did not exhibit increased nonlinear ultrasound signal (Figure S1a), indicating that phosphorylation of the engineered motif alone is not sufficient for reporter activation and that the insertion site must be carefully selected to support effective coupling to GV mechanical modulation.

Both UReKA1 and UReKA2 displayed reversible, phosphorylation-dependent modulation of nonlinear ultrasound contrast. UReKA1 showed a 4.5-fold signal increase upon PKA treatment when imaged at 350 kPa, which was fully reversed by lambda-protein phosphatase (LPP), while UReKA2 demonstrated a 12.9-fold increase when imaged at 489 kPa (Figure 1d,e). For both UReKA variants, point mutation of the Thr-to-Ala knockout (KO) mutant abolished the response, confirming that the observed modulation arose from phosphorylation of the engineered threonine. These acoustic measurements correlated with pressurized absorbance spectroscopy assays,^39^ where the midpoint collapse pressure decreased from 398 ± 43 kPa to 227 ± 20 kPa for UReKA1 and from 424 ± 64 kPa to 283 ± 15 kPa for UReKA2 upon PKA phosphorylation (Figure S1b–d). LPP coincubation restored collapse pressure to baseline, supporting the mechanical reversibility of the system. The hydrodynamic size of the biosensing GVs, as evaluated by dynamic light scattering, remained unchanged with kinase treatment (Figure S1e). The hydrodynamic diameter was 301.7 ± 4.1 nm and 543.2 ± 17.2 nm for UReKA1 and UReKA2, respectively, at baseline, with no significant change in the presence of PKA or when compared to their corresponding threonine knockouts. The larger effective diameter of UReKA2 suggests more clustering of the particles compared to UReKA1.

To assess kinase specificity *in vitro*, we incubated purified UReKA GVs with a panel of purified kinases representing both the intended target and potential off-target serine/threonine kinases. As expected, PKA treatment produced the largest increase in nonlinear ultrasound contrast, consistent with phosphorylation-dependent activation of the reporter. Exposure to other kinases, including PKG, PKCβ, PKCδ, and CaMKII, with similar consensus sequences^40,41^ produced substantially weaker or negligible changes for both nonlinear ultrasound and collapse pressure under the same assay conditions (Figure 1f,g, Figure S2a-d). These data are consistent with preferential responsiveness of the engineered substrate motif to PKA in the purified GV context. Although some basophilic kinases can share overlapping substrate preferences, the purified-enzyme assays indicate that phosphorylation-dependent acoustic activation of UReKA is strongly enriched for PKA relative to the other kinases tested.

After identifying AKAR-based UReKA1 as a robust, reversible reporter, we explored strategies to enhance sensitivity and signal dynamics. Insertion of the AKAR motif at alternative repeat positions or in multiple repeats disrupted GvpC-GV binding and failed to produce functional constructs (Figure S3a). Similarly, fusion of the phosphoamino acid-binding forkhead-associated domain 1 (FHA1)^42,43^ to AKAR-GvpC was hypothesized to amplify conformational change but instead led to protein aggregation and loss of function (Figure S3b). We also screened Kemptide sequence (LRRASLG) and modified Kemptide sequence (LRRASLP) substrates (Figure S3c,d), which showed limited responses and weaker GV reinforcement compared to AKAR-based variants. Collectively, these results establish UReKA1 and UReKA2 as kinase-responsive acoustic biosensors, exhibiting strong, reversible, and selective ultrasound readouts of enzymatic phosphorylation.

### Characterization of UReKA in Purified GVs

To establish the molecular basis of UReKA activation, we first verified the engineered phosphorylation site in the initial validated variant, UReKA1. Phos-tag sodium dodecyl sulfate–polyacrylamide gel electrophoresis (SDS-PAGE)^44^ verified phosphorylation of GvpC upon PKA treatment (Figure 2a). Tandem mass spectrometry (LC-MS/MS)^45^ further confirmed a single phosphorylated residue at Thr60 within the AKAR motif, with no other phosphorylation detected across the protein sequence (Figure 2b). Together, these data validated that PKA specifically modifies the engineered site without detectable off-target modification of native serine or threonine residues in GvpC. Although UReKA2 exhibited a larger signal dynamic range, UReKA1 was used as the biochemical prototype for subsequent mechanistic studies and phosphosite mapping because later experiments showed faster activation kinetics.

**Figure 2:**
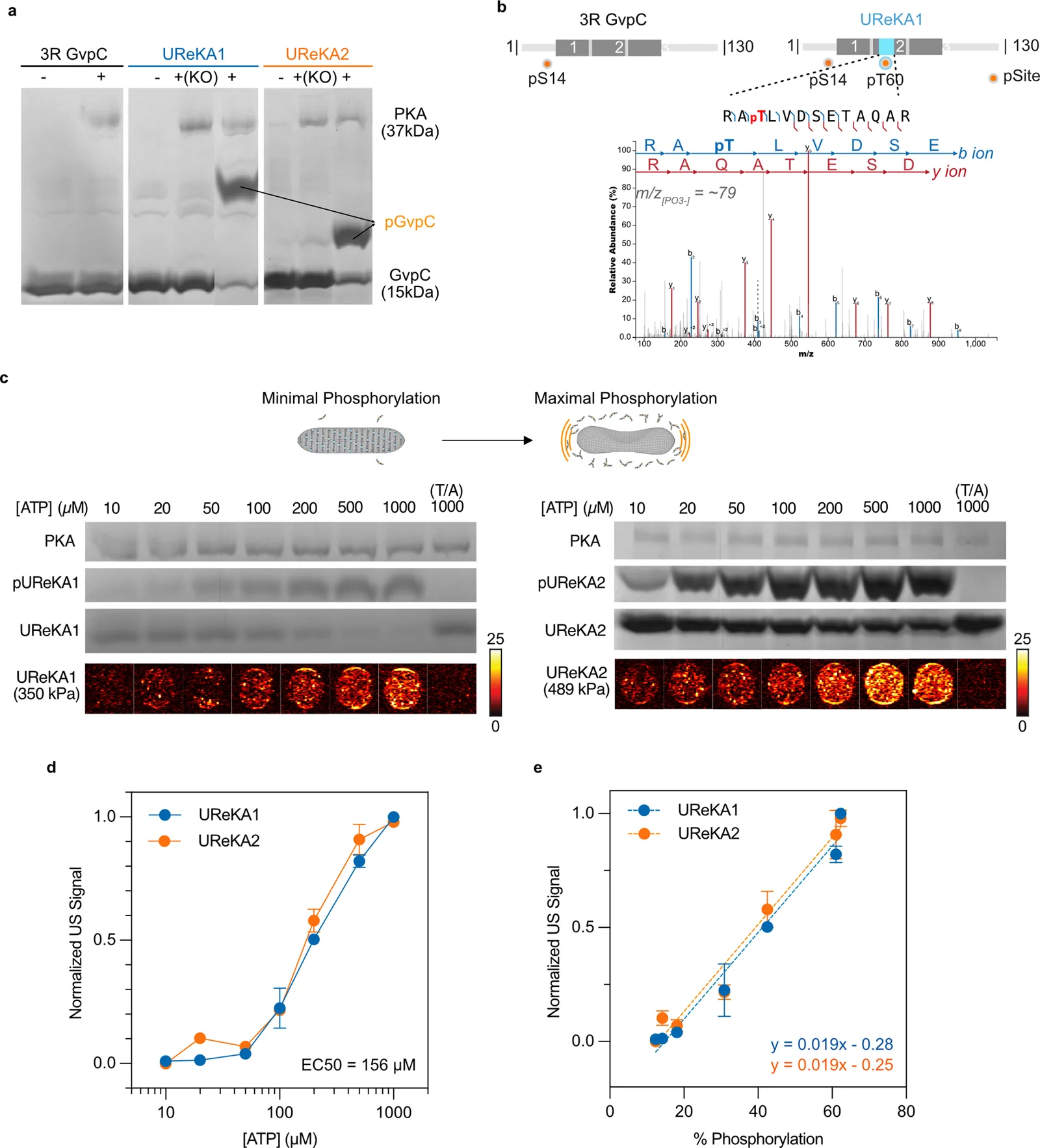
Quantitative analysis of UReKA phosphorylation. (a) Coomassie Brilliant Blue-stained SuperSep Phos-tag SDS-PAGE gel image of UReKA1 and UReKA2 with respective KO controls incubated with and without PKA. (b) Annotated LC-MS/MS spectrum of the phosphorylated peptide identified in UReKA1. Matched b- and y-ions indicated. (c) (Top) Phos-tag SDS-PAGE gel of UReKA1 and UReKA2 following incubation with increasing ATP concentrations. (Bottom) representative xAM images of UReKA1 (350 kPa) and UReKA2 (489 kPa) acquired under corresponding phosphorylation conditions. (d) Normalized nonlinear signal of UReKA1 (blue) and UReKA2 (orange) as a function of ATP concentration. Data are shown as mean ± s.d. for *n* = 3 biological replicates. Curves were fit with a 4-parameter logistic model, and the corresponding EC50 values are indicated. (e) Normalized ultrasound signal as a function of phosphorylation fraction for UReKA1 (blue) and UReKA2 (orange). For UReKA1, phosphorylation was quantified by mass spectrometry; for UReKA2, phosphorylation extent was estimated from Phos-tag gel analysis. Data are shown as mean ± s.d. (*n* = 3).

We next quantified the relationship between the degree of phosphorylation and acoustic response by titrating adenosine triphosphate (ATP) concentrations during PKA incubation (Figure 2c). As ATP concentration increased from 10 μM to 1 mM, Phos-tag SDS-PAGE revealed a progressive mobility shift consistent with increasing phosphorylation of both UReKA1 and UReKA2. In parallel, the nonlinear ultrasound contrast increased with ATP concentration, reaching saturation at 500–1000 μM. Fitting a four-parameter logistic model yielded an EC_50_ of 156 μM, well below physiological intracellular ATP levels (∼3 mM),^46^ indicating that UReKA can operate within biologically relevant regimes (Figure 2d). Parallel xAM ultrasound signal curves obtained across all ATP concentrations showed a monotonic increase in nonlinear signal amplitude with phosphorylation (Figure S4a,b).

Quantitative analysis further showed that phosphorylation scaled with acoustic signal output. For UReKA1, mass spectrometry indicated that 40 ± 5% phosphorylation of GvpC corresponded to the half-maximal ultrasound signal. For both UReKA1 and UReKA2, increasing phosphorylation was associated with an approximately linear increase in acoustic signal over the measured range (Figure 2e). Together, these data establish that UReKA contrast varies continuously with phosphorylation state, enabling a tunable acoustic readout of kinase activity in vitro.

### Characterizing Molecular Mechanism and Kinetics

Having established the specificity and reversibility of UReKA’s phosphorylation response, we next investigated the kinetic behavior and underlying molecular mechanism governing its activation. Time-course experiments revealed that the collapse pressure decreased and the nonlinear ultrasound signal increased gradually for both UReKA1 and UReKA2 following PKA incubation, reaching maximal contrast after approximately 8 and 12 h, respectively (Figure 3a,b, Figure S5a). The acoustic signal first became detectable after 2–3 h, whereas Phos-tag SDS-PAGE analysis showed that phosphorylation of the GvpC scaffold was already complete within this early period (Figure 3c,d, Figure S4a). The decoupling between phosphorylation and acoustic activation thus suggests that the rate-limiting step in UReKA response is not the enzymatic modification itself but either a slower structural transition or dissociation process that follows phosphorylation. Because phosphorylation of the engineered substrate occurred more rapidly than the emergence of the full acoustic response, all measurements were acquired at end point after extended incubation of over 12 h unless it was specifically a time-course experiment, such that the reported ultrasound signals reflect the accumulated phosphorylation-dependent mechanical state rather than instantaneous kinase activity.

**Figure 3:**
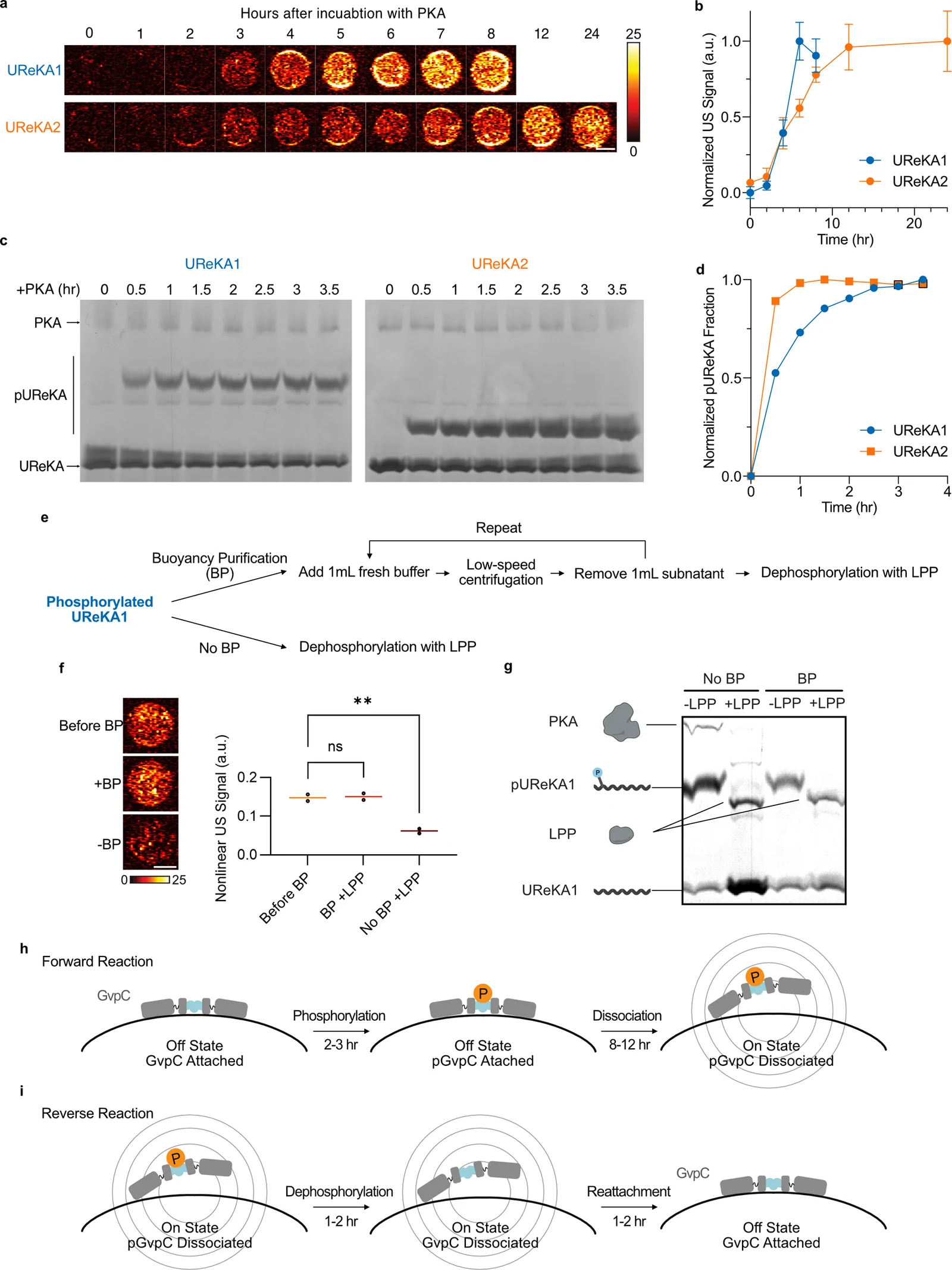
Kinetics and molecular mechanism of UReKA. (a) xAM images of UReKA1 and UReKA2 incubated with PKA at various time points at 634 kPa (for maximal signal). (b) Normalized nonlinear US signal of UReKA1 (blue) and UReKA2 (orange) over time (*n* = 3). (c) Coomassie Blue-stained Phos-tag SDS-PAGE image of UReKA (bottom band) at various incubation time points with PKA (top band). Band intensity between the top and bottom bands indicates the phosphorylated fraction of UReKA GvpC. (d) Normalized phosphorylated fraction of UReKA1 (blue) and UReKA2 (orange) over time (*n* = 1). (e) Workflow schematic (top) describing buoyancy purification (BP) of phosphorylated UReKA1 followed by dephosphorylation with LPP. (f) Nonlinear ultrasound images (left) and signal (right) of UReKA1 incubated with PKA and phosphorylated UReKA1 incubated with LPP with or without buoyancy purification (*n* = 2). (g) Phos-tag SDS-PAGE gel showing band shifts corresponding to phosphorylated (pUReKA1) and dephosphorylated states under different conditions (No BP, BP, ± LPP). (h, i) Schematics of the proposed molecular mechanism of UReKA with reversible phosphorylation reaction, with phosphorylation as the forward reaction (h) and dephosphorylation of GvpC as the reverse reaction (i).

Due to its superior temporal response, UReKA1 was chosen for a more detailed kinetic characterization, while UReKA2 remains a candidate for future applications requiring greater dynamic range. To distinguish whether the signal enhancement arises from partial structural rearrangement or full detachment of phosphorylated GvpC from the gas-vesicle surface, we performed a buoyancy purification (BP) assay to separate the buoyant GV-bound fraction from any unbound GvpC (Figure 3e). When phosphorylated UReKA1 samples were incubated with LPP to induce dephosphorylation, both nonlinear ultrasound signal and collapse pressure were restored to baseline, consistent with previously shown data (Figure 3f, Figure S5b,c). In contrast, when LPP was incubated with phosphorylated UReKA1 post-BP—without any dissociated fraction of GvpC—the ultrasound signal remained elevated and indistinguishable from phosphorylated controls, indicating that the phosphorylated GvpC fraction had been cleared via the purification. Moreover, a significant loss of the phosphorylated UReKA1 GvpC band was observed from Phos-tag SDS-PAGE following BP (Figure 3g). While initial observations suggested either a structural rearrangement or dissociation of GvpC, these data support dissociation as the dominant mechanism.

From these observations, we propose a two-step molecular mechanism for UReKA activation (Figure 3h,i). In the forward reaction, phosphorylation of GvpC by PKA introduces negative charge that weakens electrostatic interactions with the GV surface, promoting dissociation and generating the acoustically “on” state. In the reverse reaction, dephosphorylation by LPP allows unmodified GvpC to reassociate with the GV shell, restoring mechanical rigidity and the acoustically “off” state. The observed delay between phosphorylation and acoustic activation therefore reflects the kinetics of protein–shell dissociation and reattachment, rather than chemical turnover at the phosphorylation site.

Altogether, these results establish that UReKA functions through a phosphorylation-triggered GvpC detachment mechanism, where enzyme-controlled modulation of GvpC-shell binding governs the mechanical and acoustic states of the reporter. This mechanistic understanding provides a foundation for further tuning of the sensor response speed and reversibility through protein-interface engineering.

### In Cellulo Response of UReKA

Having validated UReKA’s reversible and quantitative response to phosphorylation in vitro, we next sought to demonstrate its functionality as an intracellular kinase activity sensor in mammalian cells. We selected UReKA1 as the prototype variant for in cellulo expression owing to its faster activation kinetics compared to UReKA2. To enable heterologous GV formation and UReKA expression, we employed a two-plasmid system in human embryonic kidney 293 cells expressing the SV40 Large T antigen (HEK293T) (Figure 4a). The first plasmid encoded the GV structural operon (gvpNJKFGWV) under a CMV promoter, while the second plasmid carried the gas vesicle protein A (gvpA) gene and the UReKA-modified gvpC gene, linked by an internal ribosome entry site (IRES)^47^ element to control relative expression levels.^25^ This modular configuration allowed fine-tuning of the GvpA:GvpC stoichiometry, which we previously identified as a critical determinant of UReKA activation dynamics due to the rate-limiting GvpC dissociation step.

**Figure 4:**
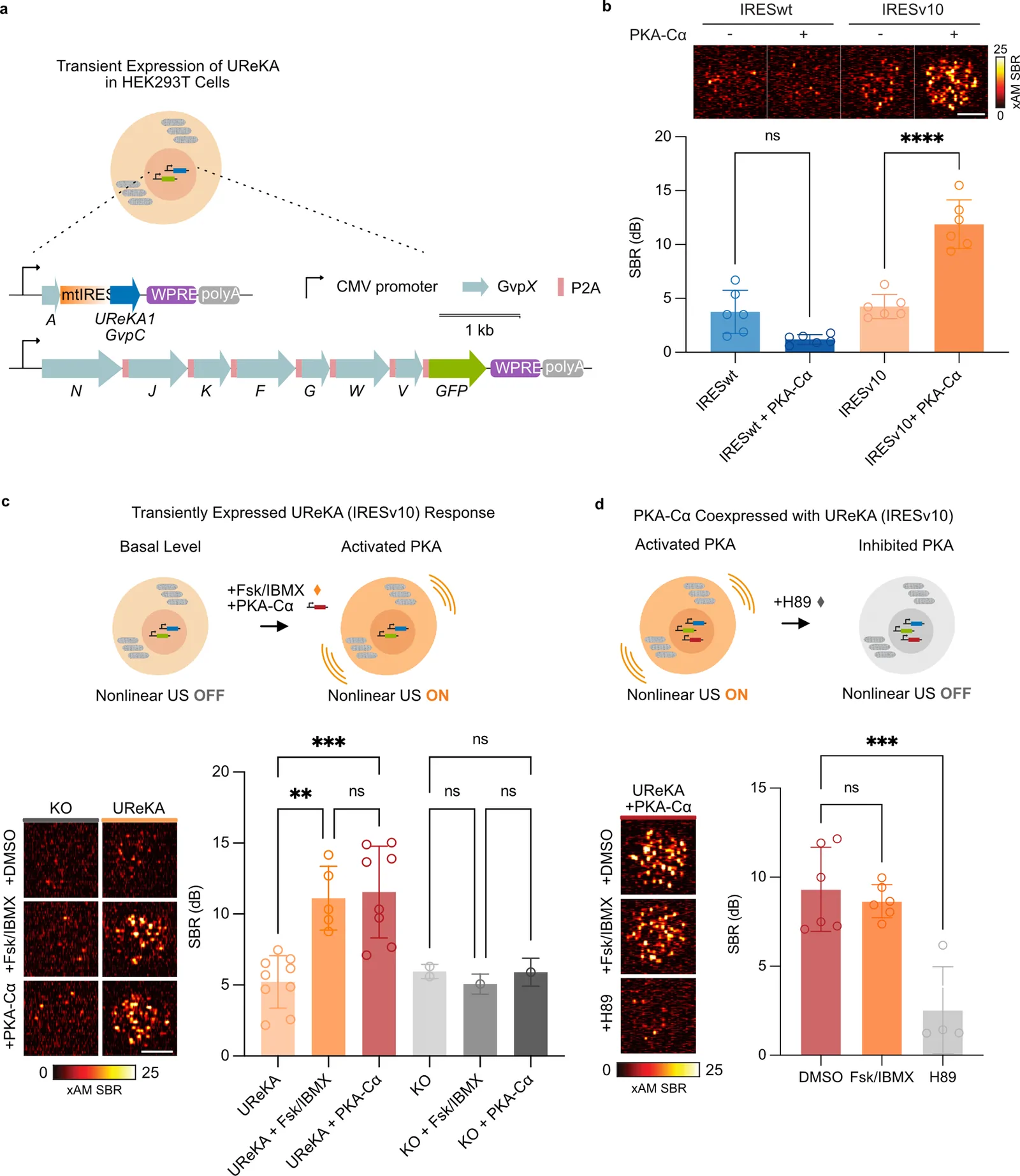
Pharmacological response of UReKA-GVs in HEK293T. (a) Schematics of the genetic constructs for transient expression of UReKA in HEK293T cells. (b) (Top) Representative nonlinear xAM image at 419 kPa of UReKA GVs with IRESwt and IRESv10 upstream of GvpC with or without coexpression of PKA-Cα catalytic subunit and (bottom) respective SBR; *n* = 6. (c) (Top) Schematics of applying UReKA-Iv10 to image intracellular PKA activation through either FSK/IBMX stimulation or coexpression of PKA-Cα catalytic subunit. (Bottom) xAM SBR (left) and representative image (right) of UReKA-Iv10 with FSK/IBMX stimulation or PKA-Cα coexpression at 419 kPa. T/A mutant was used as the KO control, and DMSO was used as the vehicle control. Each data point indicates a biological replicate. (d) (Top) Schematics of transiently expressed UReKA-Iv10 coexpressed with PKA-Cα applied to image intracellular PKA inhibition with H89. (Bottom) xAM SBR (left) and representative image (right) of UReKA-Iv10 with FSK/IBMX or H89 stimulation at 419 kPa. DMSO was used as the vehicle control. Each data point indicates a biological replicate.

To optimize the GV–GvpC balance, we screened a gradient of IRES mutant variants (IRESv10–v12) spanning a range of translation efficiencies^48^ (Figure S6a). Among these, the IRESv10 variant provided an optimal dynamic range, with sufficient GvpC to suppress UReKA activation under baseline conditions. Constructs using IRESv10 displayed markedly higher nonlinear ultrasound signal upon PKA activation compared to wild-type IRES, while maintaining minimal basal background (Figure 4b). Consequently, the UReKA1 with the IRESv10 mutant variant was selected for all subsequent mammalian experiments.

We next evaluated UReKA’s response to intracellular modulation of PKA activity. Cells expressing UReKA1 were stimulated with a combination of forskolin (FSK),^49–51^ an adenylate cyclase activator, and 3-isobutyl-1-methylxanthine (IBMX),^51^ a phosphodiesterase inhibitor, both of which elevate intracellular cyclic adenosine monophosphate (cAMP) levels and thereby activate endogenous PKA signaling (Figure 4c). Alternatively, to mimic pathological PKA hyperactivation,^52^ cells were cotransfected with a PRKACA construct encoding a constitutively active PKA catalytic subunit (PKA-Cα_L206R_). In both conditions, UReKA-expressing cells exhibited robust increases in nonlinear ultrasound contrast, with 5–10 dB higher signal-to-background ratio (SBR) compared with vehicle-treated or kinase-inactive controls. Notably, activation of endogenous PKA signaling with FSK/IBMX was sufficient to produce a strong acoustic response even without kinase overexpression, indicating that UReKA can report changes in pathway activity under native expression conditions. For all cellular xAM measurements, imaging was performed at an applied acoustic pressure of 419 kPa. An acoustic pressure ramp on cellular samples (Figure S6b) showed that the nonlinear signal of activated UReKA increased with applied pressure and plateaued without decline through 939 kPa, confirming that GVs are not acoustically collapsed at the 419 kPa imaging pressure. This pressure was selected to provide sufficient nonlinear signal-to-background in the heterogeneous cellular environment, where lower pressures compressed the dynamic range needed to resolve activation states.

Conversely, treatment with the PKA inhibitor H89 ^53^ effectively reversed the signal enhancement (Figure 4d), reducing the nonlinear response to baseline levels even in cells overexpressing PKA-Cα. These results indicate that UReKA primarily reports the phosphorylation state of the reporter pool, and thus the net catalytic output of PKA activity, rather than kinase abundance alone. Together, these data show that elevated kinase expression is not sufficient to sustain reporter activation when catalytic activity is inhibited, whereas activation of endogenous signaling is sufficient to drive a robust acoustic response.

To further assess cellular specificity, we tested whether activation of other signaling pathways was sufficient to induce UReKA signal in the absence of direct PKA stimulation. Cells were treated with phorbol 12-myristate 13-acetate (PMA) to activate PKC-associated signaling,^54–56^ ionomycin or thapsigargin to elevate intracellular calcium^57^ and engage calcium-dependent signaling pathways,^58^ and insulin-like growth factor 1 (IGF-1) to stimulate growth factor-linked signaling.^59^ Notably, UReKA-expressing cells treated with PMA produced a robust UReKA response comparable to FSK/IBMX and PKA-Cα overexpression that was also reversible by the PKA inhibitor H89 blocking control (Figure S7a,b). Because PMA is known to activate PKC-associated pathways and has also been reported in some contexts to elevate cAMP levels in HEK293 cells,^56^ this result may reflect indirect activation of PKA signaling rather than direct phosphorylation by PKC. Therefore, while UReKA does not respond broadly to all non-PKA stimuli tested, these data highlight that cellular specificity is pathway-dependent and cannot be inferred solely from substrate design.

An additional practical consideration for genetically encoded acoustic reporters is whether the expression of GVs perturbs cell viability or physiology. We did not observe an obvious reduction in cell viability through the CCK-8 assay^60^ in UReKA-expressing HEK293T cells incubated with the FSK/IBMX complex, H89, or overexpression of PKA-Cα over 72 h relative to controls (Figure S8). Although more detailed studies will be required to assess potential effects on specific signaling pathways or longer-term cellular states, these results support the general compatibility of the current interrogation conditions.

More broadly, because the present cellular experiments were analyzed at end point after prolonged stimulation or kinase overexpression, the measured UReKA signal should be interpreted as the accumulated phosphorylation-dependent state of the reporter rather than a real-time readout of reversible GvpC exchange. In this setting, transient intracellular turnover of phosphorylated GvpC is unlikely to substantially affect the interpretation of the current data, although it will likely become important for future longitudinal or fully reversible implementations.

Overall, this system establishes UReKA as a genetically encodable, enzyme-responsive acoustic reporter capable of resolving kinase activity states within living mammalian cells. Importantly, the cellular perturbation experiments indicate that the UReKA readout reflects net pathway activity rather than enzyme expression level alone. The tunable IRES-based design further provides a generalizable strategy for balancing the stoichiometric expression of structural and functional protein components in acoustogenetic constructs.

## Conclusions

In this study, we introduced UReKA as an acoustic biosensor of kinase activity. Using purified recombinant GVs, we demonstrated that UReKA specifically responds to phosphorylation at engineered sites by the targeted kinase, with these modifications reversibly changing the GV’s nonlinear acoustic contrast. We also confirmed that the observed nonlinear signal activation is driven by detachment of GvpC from the surface of the GVs, similar to the ultrasonic reporter of calcium (URoC) system.^28^ Additionally, we showed that UReKA expressed in mammalian cells effectively detects intracellular PKA activity, exhibiting increased or decreased signal in response to kinase activation or inhibition through pharmacological stimulation or genetic modification. Taken together, these results lay the groundwork for an ultrasonic reporter system for kinases that is quantitative and reversible.

The *in cellulo* applications demonstrated in this work suggest that UReKA has strong potential for future use in drug screening, especially for kinase inhibitors, and kinase activity imaging in live animals for cancer and neurobiological research. The potential ability to monitor real-time kinase dynamics and pharmacological responses to novel therapeutics could provide valuable insights into cellular signaling pathways and drug mechanisms of action. Furthermore, complementary to fluorescence- and positron emission tomography (PET)-based reporters, UReKA’s acoustic signal readout could in the future enable deep-tissue imaging and real-time molecular monitoring, offering a unique advantage for studying dynamic cellular signaling in its native physiological context.

While the genetic constructs described in this study could be applied to some experimental settings, further optimization is required to enhance UReKA’s adaptability and suitability for live-monitoring sensors both *in cellulo* and *in vivo*. In its current implementation, UReKA is best interpreted as an end point or time-integrated reporter that does not directly report instantaneous kinase activity, because phosphorylation occurs more rapidly than the full acoustic response develops. Moreover, the current preparation of the cells for ultrasound measurement requires dissociation, concentration matching, and embedding in agarose phantoms, a process that takes time comparable to the dephosphorylation kinetics resolving over roughly 1–2 h. This makes the current implementation well suited for sustained perturbations, drug-response profiling, and comparisons between relatively stable signaling states, but less suitable for capturing second- to minute-scale signaling transients, pulsatile kinase activity, or other rapidly reversible signaling events in living cells or *in vivo*. Moreover, while purified-enzyme assays demonstrate strong selectivity for PKA, cellular specificity depends on pathway context and may reflect indirect activation mechanisms.

One key area for future improvement is therefore to accelerate coupling between phosphorylation and GV mechanical modulation so that UReKA can more faithfully track transient kinase dynamics. Following the examples from generations of AKARs that improved over the past couple of decades, future work may improve UReKA through rational design, directed evolution, and computational modeling of specific substrate and transduction elements.^61,62^ For example, a design that does not rely on complete GvpC dissociation may enable faster turnover between phosphorylation states and improve the responsiveness to transient kinase activity. In cellular settings, effective reversibility will likely depend on the coupled kinetics of GvpC phosphorylation, dissociation, intracellular turnover, replenishment, and rebinding to the GV surface. This consideration also motivated the IRES-based expression design used here to tune the stoichiometric balance between GVs and engineered GvpC, maintaining sufficient baseline coating while avoiding excess free GvpC that could rapidly replace detached molecules and reduce the phosphorylation-dependent contrast.

Although purified GV measurements established a linear relationship between the phosphorylation extent and acoustic output *in vitro*, direct quantitative calibration of the cellular UReKA signal to absolute kinase activity remains challenging. In cells, the measured acoustic response is expected to depend not only on the phosphorylation state of the reporter but also on factors such as reporter expression level, GV abundance, cell-to-cell heterogeneity, and background tissue properties. Accordingly, in the present study, the cellular readout should be interpreted primarily as a relative indicator of kinase pathway activation state rather than an absolute measure of enzymatic activity. Future work will require normalization strategies and orthogonal biochemical measurements to relate in-cell acoustic output more directly to absolute kinase activity and to establish graded pharmacological dose–response relationships.

Additionally, although this study demonstrates robust signal dynamics *in cellulo*, validation in more physiologically relevant systems such as tissue models, organoids, or *in vivo* animal settings will be an important next step toward translation. The present study was intentionally focused on purified preparations and cultured mammalian cells to establish the molecular mechanism and intracellular functionality of the ultrasonic biosensor of kinases under controlled conditions before addressing the added complexity of heterogeneous tissues and whole organisms.

Over the time frame relevant to this study, GVs remained detectable in cells for up to 72 h and retained nonlinear ultrasound contrast, consistent with the persistence of a functionally active gas-filled reporter population. Looking forward, deployment of UReKA in more complex biological settings could leverage gene-delivery strategies commonly used for genetically encoded reporters, including viral vectors and inducible or cell-type-specific expression systems. As a near-term step toward *in vivo* application, engineered cells expressing UReKA could be evaluated in engraftment models, enabling noninvasive longitudinal monitoring of kinase activity in a physiologically relevant context. Translation to intact tissues and whole animals will additionally require optimization of imaging geometry, acoustic pressure delivery, and reporter expression level to accommodate attenuation, tissue heterogeneity, and depth-dependent nonlinear signal detection.

Another important direction will be to extend UReKA variants for additional target kinases, including PKC, Akt, and receptor tyrosine kinases (RTKs), by replacing the engineered phosphorylation motif and optimizing reporter sensitivity and specificity for each signaling context. Supporting the modularity of this design, supplementary screening identified additional PKA-responsive variants incorporating alternative substrate-sequence designs, suggesting that substrate-level engineering can preserve the phosphorylation-dependent acoustic output. Such advances could enable broader and more reliable signal detection across diverse applications in biology and medicine.

Overall, UReKA represents an approach to ultrasound-based molecular imaging for post-translational protein modification, providing a noninvasive and reversible platform and, in purified systems, quantitative monitoring of kinase activity states. This advance offers a “Eureka” moment for molecular imaging of live-cell signals in opaque media.

## Materials and Methods

### Design and Cloning of Genetic Constructs

All plasmids used in this study were constructed using a combination of polymerase chain reaction (PCR) with Q5 polymerase and either Gibson Assembly^63^ or KLD mutagenesis, using enzymes from New England Biolabs (NEB) and custom primers from Integrated DNA Technologies (IDT). For screening and characterization of purified proteins, all *gvpC* gene sequences were codon-optimized for *E. coli* expression and introduced by substitution-insertion into the second repeat of the WT *gvpC* gene sequence in a pET28a expression vector (Novagen) driven by a T7 promoter and lac operator. Specifically, the construct encoding the three-repeat *Ana gvpC* gene was generated by deleting the fourth and fifth repeats from the WT *Ana gvpC* sequence while appending a C-terminal 6xHis tag (Addgene no. 85732) via KLD mutagenesis. Subsequently, codon-optimized PKA recognition motifs, including AKAR (LRRATLVD), emptide (LRRASLG), or modified Kemptide (LRRASLP) sequences, were introduced through KLD mutagenesis to generate UReKA GvpC variants. Additional modifications, such as FHA1 fusion or SUMOylation-tag insertion, were achieved through Gibson Assembly.

For transient expression of UReKA in mammalian cells, all sequences were codon-optimized for human cell expression and cloned into a pCMV-Sport vector with WPRE-hGH polyA driven by a CMV promoter. Similar modifications were made to the WT *gvpC* sequence to generate UReKA1 and knockout-control *gvpC* constructs, which were then inserted into an existing plasmid encoding the *gvpA* gene (Addgene #197588), preceded by an IRES sequence, using Gibson Assembly. An existing plasmid was used for the expression of all seven other chaperone proteins (*gvpNJKFGWV*) required for GV formation (Addgene #197589). For constitutive PKA activation, a gBlock encoding the *PRKACA* gene (NM_002730.4) was custom ordered from IDT and inserted into the backbone plasmid via Gibson Assembly.

### Expression and Purification of UReKA Gas Vesicles

For *in vitro* purified protein assays, GVs were collected and purified from confluent *Ana* cultures following previously published protocols.^29,39^ Briefly, *Ana* cells were grown in Gorham’s medium supplemented with BG-11 solution (Sigma-Aldrich) and 10 mM sodium bicarbonate at 25 °C under 1% CO_2_ and 100 rpm shaking, with a 14 h light/10 h dark cycle. Confluent cultures were transferred to sterile separating funnels and left undisturbed for 2–3 days, allowing buoyant *Ana* cells expressing GVs to float, and the subnatant was drained. GVs were released by hypertonic lysis using 10% SoluLyse (Amsbio L200125) and 500 mM sorbitol. Purified GVs were obtained through 3–4 rounds of buoyancy purification at 300 g for 4 h per round, where, after each round, the subnatant was removed and the pellet was resuspended in 6 M urea solution buffered with 100 mM Tris-HCl (pH 8–8.5) to strip the native outer layer of GvpC. After urea treatment, GVs were either maintained in 6 M urea for subsequent GvpC readdition or dialyzed in 4 L PBS (Corning) for at least 12 h at 4 °C for long-term storage. For dialysis, the stripped GVs were loaded into regenerated cellulose dialysis pouches with a 6–8 kDa molecular weight cutoff (Spectrum Laboratories).

The engineered GvpC variants were expressed and purified following previously established protocols.^29,39^ For protein expression, UReKA GvpC variants were transformed into chemically competent BL21­(DE3) cells (Invitrogen) and grown overnight at 37 °C for 12–16 h in 5 mL starter cultures of 2xYT medium supplemented with 50 μg/mL kanamycin. The starter cultures were diluted 1:100 into autoinduction Terrific Broth (Novagen 71491) with 50 μg/mL kanamycin and incubated at 30 °C with shaking at 250 rpm for 20–24 h to induce protein expression. Cells were then harvested by centrifugation at 5000 rcf and lysed at room temperature using SoluLyse (Amsbio L200125) supplemented with lysozyme (400 μg/mL) and DNase I (10 μg/mL). GvpC inclusion bodies were isolated by centrifugation at 13,000 rcf for 10 min, resuspended in solubilization buffer (20 mM Tris-HCl, 500 mM NaCl, 6 M urea, pH 8.0), and incubated with Ni-NTA resin (QIAGEN) for 2 h at 4 °C for His-tag purification. Wash and elution buffers contained the same composition as the solubilization buffer, but with 20 mM and 250 mM imidazole, respectively. The concentration of purified protein was determined using the Bradford reagent (Bio-Rad), and >95% purity was verified by SDS-PAGE analysis.

The concentration of Ana GVs was determined by measuring their optical density (OD) at 500 nm (OD_500_) using a Nanodrop spectrophotometer (Thermo Fisher Scientific), with the resuspension buffer as a blank. Based on previous work, a GV concentration of OD_500_ = 1 corresponds to ∼184 pM with a gas fraction of 0.0417%. GvpC variants, expressed and isolated from inclusion bodies, were added to stripped *Ana* GVs in 6 M urea in 2–3 fold molar excess after accounting for the 1:15 binding ratio of 3-repeat GvpC:GvpA. The precise quantity of recombinant GvpC (in nanomoles) required for a 2-fold stoichiometric excess was calculated using the formula: 2 × OD_500_ × 480 nM × volume of GVs (in liters). Recombinant GvpC was refolded onto the surface of the stripped GVs by dialysis in 4 L PBS at 4 °C for at least 12 h. Dialyzed GV samples underwent two or more rounds of buoyancy purification at 300*g* for 3–4 h to remove excess unbound GvpC, during which, after each round of centrifugation, the sample buffer was exchanged into kinase reaction buffer (50 mM Tris-HCl, 10 mM MgCl_2_, 0.1 mM EDTA, pH 7.5) for further characterization.

### In Vitro Phosphorylation Assay

For phosphorylation assays, 250 units of PKA (P6000L; NEB; specific activity: 1 pmol/min/unit), 1.5 μg of human CaMKIIα (Sino Biological; specific activity: 160 nmol/min/mg), 0.25 μg of active human PRKG1 (Sino Biological; specific activity: 890 nmol/min/mg), 1 μg of active PKCδ (Sino Biological; specific activity: 250 nmol/min/mg), or 1 μg of PKCβ (ThermoFisher; specific activity: 320 nmol/min/mg) per μL of engineered GVs at OD_500_ = 10 were mixed with 1 mM ATP (Sigma-Aldrich) in NEBuffer for Protein Kinases (50 mM Tris-HCl, 10 mM MgCl_2_, 0.1 mM EDTA, 2 mM DTT, 0.01% Brij 35, pH 7.5; NEB) and incubated with slow rotation at 37 °C for 10–12 h, unless otherwise specified. For dephosphorylation assays, 10 units of LPP (P0753L; NEB) per μL of engineered GVs at OD_500_ = 10 were mixed with PMP Buffer (50 mM HEPES, 100 mM NaCl, 2 mM DTT, 0.01% Brij 35, pH 7.5) and 1 mM MnCl_2_, followed by incubation with slow rotation at 37 °C for 10–12 h.

### Pressurized Absorbance Spectroscopy

For initial screening of UReKA variants, collapse pressure was determined using previously established protocols.^29,39^ Briefly, purified Ana GVs were diluted in experimental buffers to an OD_500_ of approximately 0.2–0.4, and 400 μL of the diluted sample was loaded into a flow-through quartz cuvette with a 1 cm path length (Hellma Analytics). Hydrostatic pressure was applied using a 1.5 MPa nitrogen gas source, regulated via a single-valve pressure controller (PC series; Alicat Scientific). Optical density (OD_500_) was continuously measured using a microspectrometer (STS-VIS; Ocean Optics) as pressure increased from 0 to 1 MPa in 50 kPa increments, with a 7-s equilibration period between each measurement. Each data set was normalized by scaling to the min-max measurement values, and data were fitted using the Boltzmann sigmoid function $f\left(P\right)={\left(1+e^{\left(p-p_{c}\right)/\Delta p}\right)}^{-1}$ , where *p*_c_, the collapse pressure, represents the midpoint of normalized OD change. The 95% confidence intervals (CIs) were rounded to the nearest integer and are reported in the figures.

### Denaturing Polyacrylamide Gel Electrophoresis (SDS-PAGE and Phos-tag Gels)

GV samples incubated with PKA or LPP were concentration-matched at OD_500_ = 10 and mixed 1:1 with 2× Laemmli buffer (Bio-Rad), containing SDS and 2-mercaptoethanol. The samples were then boiled at 95 °C for 5 min, and 20 μL of the samples were loaded into either a premade polyacrylamide gel (Bio-Rad) or Phos-tag Gel (Fujifilm) immersed in 1× Tris-Glycine-SDS buffer. In regular SDS-PAGE gels, 10 μL of Precision Plus Protein Dual Color Standards (Bio-Rad) was loaded as the ladder. Electrophoresis was performed at 120 V for 55 min, after which the gel was washed in DI water for 15 min to remove excess SDS and Coomassie-stained for 1 h on a rocker using SimplyBlue SafeStain (Invitrogen). The gel was allowed to destain overnight in DI water before imaging using a Bio-Rad ChemiDoc imaging system.

### Mass Spectrometry

Proteins were denatured in a buffer containing 8 M urea (U0631, Sigma-Aldrich) and 100 mM Tris, pH 8.5. The sample was further reduced by incubation with 10 mM tris­(2-carboxyethyl)­phosphine (TCEP, C4706, Sigma) at 37 °C for 20 min, then alkylated by incubation with 10 mM 2-chloroacetamide (CAA, 79-07-2, Millipore Sigma) in the dark at room temperature for 15 min. Subsequently, the sample was digested with 0.1 μg LysC (125-05061, Wako) at 37 °C for 4 h, diluted 4-fold with 100 mM Tris, pH 8.5, and CaCl_2_ was added to 1 mM. The sample was then digested with 0.2 μg of trypsin (90305, Thermo Fisher Scientific) overnight at 37 °C. Samples were acidified by adding trifluoroacetic acid to 0.5% and then desalted using Pierce C18 spin columns (89868, Thermo Scientific). After desalting, the peptides were dried and reconstituted in water containing 0.2% formic acid (FA, A11750, Fisher Scientific) and 2% acetonitrile (ACN, A9554, Fisher Scientific) for subsequent LC-MS/MS analysis.

LC-MS/MS analysis was performed with an EASY-nLC 1200 (ThermoFisher Scientific, San Jose, CA) coupled to a Q Exactive HF hybrid quadrupole-Orbitrap mass spectrometer (ThermoFisher Scientific, San Jose, CA). Peptides were separated on an Aurora UHPLC column (25 cm × 75 μm, 1.6 μm C18, AUR2–25075C18A, Ion Opticks) with a flow rate of 0.35 μL/min for a total duration of 43 min and ionized at 1.7 kV in the positive ion mode. The gradient was composed of 6% solvent B (2 min), 6–25% B (20.5 min), 25–40% B (7.5 min), and 40–98% B (13 min); solvent A: 2% ACN and 0.2% FA in water; solvent B: 80% ACN and 0.2% FA. MS1 scans were acquired at a resolution of 60,000 from 375 to 1500 *m*/*z*, with an AGC target of 3e6, and a maximum injection time of 15 ms. The 12 most abundant ions in MS1 scans were selected for fragmentation via higher-energy collisional dissociation (HCD) with a normalized collision energy (NCE) of 28. MS2 scans were acquired at a resolution of 30,000, with an AGC target of 2e5 and a maximum injection time of 120 ms. Dynamic exclusion was set to 30 s, and ions with charges of +1, + 7, + 8, and >+8 were excluded. The temperature of the ion transfer tube was 275 °C, and the S-lens RF level was set to 55.

RAW files were searched with Proteome Discoverer SEQUEST (version 2.5, Thermo Scientific) against the FASTA file containing Ana GvpC, GvpA, and mutant GvpC protein sequences. Trypsin/P was set as the digestion enzyme, allowing for a maximum of two missed cleavages. Dynamic modifications were set to oxidation on methionine (M, + 15.995 Da), deamidation on asparagine and glutamine (N and Q, + 0.984 Da), phosphorylation on serine, threonine, and tyrosine (S, T, and Y, + 79.966 Da), and protein N-terminal acetylation (+42.011 Da) and Met loss (−131.040 Da). Carbamidomethylation on cysteine residues (C, + 57.021 Da) was set as a fixed modification. The maximum parental mass error was set to 10 ppm, and the MS2 mass tolerance was set to 0.05 Da. The relative abundance of parental peptides was calculated by integration of the area under the curve of the MS1 peaks using the Minora LFQ node. The maximum false peptide discovery rate was specified as 0.01 using the Percolator node, validated by q-value. The IMP-ptmRS node within Proteome Discoverer was used to calculate PTM site probabilities. The RAW data have been deposited to the ProteomeXchange Consortium via the PRIDE partner repository with the data set identifier PXD071157.^64^ Spectral annotation was generated by the Interactive Peptide Spectral Annotator (IPSA).^65^

### DLS Measurements

Following previously established protocols,^29,39^ engineered *Ana* GVs were diluted to an OD_500_ of approximately 0.2 in experimental buffers. Then, 1 mL of the sample was loaded into a disposable cuvette, and particle size was measured using the ZetaPALS particle sizing software (version 4.0, Brookhaven Instruments Corporation) with an angle of 90° and a refractive index of 1.33.

### HEK293T Cell Culture and Transient Transfection

HEK293T cells (American Type Culture Collection (ATCC), CLR-3216) were seeded in 12-well plates at a density of 1.5 × 10^5^ cells per well and maintained at 37 °C with 5% CO2 in a humidified incubator. Cells were cultured in 1 mL of DMEM (Corning, 10-013-CV) with 10% FBS (Gibco), 10 mM HEPES (Cytiva), and 1× penicillin–streptomycin 24 h before transfection. Transient transfection mixtures were created by mixing 2 μg of plasmid mixture with Transporter 5 (Polysciences 26008) at a 1:4 DNA/Transporter ratio. The mixture was incubated for 20 min at room temperature and added dropwise to HEK293T cells. Media were changed after 12–16 h and daily thereafter. For the transient expression of UReKA and dUReKA, the mutant control, 280 fmol of the *gvpA*-IRES-*gvpC* plasmid and 70 fmol of the plasmid encoding *gvpNJKFGWV* were added into the DNA mixture, and pUC19 plasmid DNA was supplemented to make the total amount of DNA 2 μg. The gvpA-IRESv10-gvpC and gvpNJKFGWV constructs are 7,518 bp and 10,484 bp, respectively (Table S1); the 280 fmol and 70 fmol correspond to approximately 1.37 μg and 0.48 μg (a 4:1 molar ratio favoring the gvpA-containing cassette). For the PKA activation study, either 35 fmol of *PRKACA* was added to the DNA mixture to overexpress the catalytic subunit or the media were supplemented with 20 μM forskolin (MedChemExpress) and 500 μM 3-isobutyl-1-methylxanthine (Sigma-Aldrich) 12 h prior to harvesting the cells to saturate the PKA activity level. For the PKA inhibition study, 50 μM H89 dihydrocloride (Tocris) was supplemented to the media for at least 3 h. Transfected cells were assayed 72 h after transfection. For other kinase stimulation study, 100 nM PMA (MedChemExpress), 1 μM ionomycin, 1 μM thapsigargin (MedChemExpress), or 100 ng/mL IGF-1 (MedChemExpress) was supplemented to the media for 12 h.

To assess cell viability, Cell Counting Kit-8 (APExBio) was added to each sample well at a 1:10 dilution and incubated for 1 h. Absorbance at 450 nm was measured using a microplate reader (Tecan) and then calculated as [(A_s_ - A_b_)/(A_c_ - A_b_)] × 100%, where A_s_ is the absorbance of the sample, A_b_ is the blank media, and A_c_ is the control well with no cellular stimulation or transfection.

### Purified GV and In Vitro Ultrasound Imaging

After 3 days of expression, cells were dissociated using Trypsin/EDTA (Corning 25-053-CI), centrifuged at 300 rcf for 6 min at room temperature, and resuspended in PBS. The cells were counted using an automated cell counter (Countess 3, Thermo Fisher), and all samples were concentration-matched to 10 million cells per milliliter in PBS. Imaging phantoms were prepared with 1% agarose (w/v, Lonza, #50070) in PBS. For ultrasound imaging, either purified GVs or cells were diluted 1:1 with 1% agarose to result in a final concentration of OD1 GVs or 5 million cells per milliliter, respectively, before loading into their respective phantoms or injected into acoustically transparent tubing for continuous kinetics experiments. All samples were placed in the same buffer as the phantom during the imaging session. For kinetic measurements, the samples were maintained at 37°C by a custom water bath.

Imaging was performed using a Verasonics Vantage programmable ultrasound scanning system and a L22-14vX 128-element linear array Verasonics transducer, with a specified pitch of 0.1 mm, an elevation focus of 8 mm, an elevation aperture of 1.5 mm, and a center frequency of 18.5 MHz with 67% −6 dB bandwidth. For nonlinear image acquisition, a custom cross-amplitude modulation (xAM) sequence detailed in an earlier study^31^ was used with an xAM angle (θ) of 19.5°, an aperture of 65 elements, and a transmitting frequency of 15.625 MHz. The center of the sample wells was placed at a depth of 5 mm with a conventional ray-line scanning B-mode pulse sequence with parabolic focusing at 10 mm and an aperture of 40 elements. The focus was set far from the sample position to reduce the acoustic pressure and avoid collapsing the samples. The transmitted pressure at the sample position at 5 mm was calibrated using a Precision Acoustics fiber-optic hydrophone system. Each image was an average of 50 accumulations.

B-mode images were acquired at a transmit voltage of 1.6 V (50 kPa), and an automated voltage ramp imaging script (programmed in MATLAB) was used to conduct xAM acquisitions at different acoustic pressures. For all samples used for characterization, an xAM voltage ramp sequence from 3 V (219 kPa) to 8 V (939 kPa) in 0.5 V step increments was used. Samples were subjected to complete collapse at 25 V with the B-mode sequence for 10 s, and the subsequent postcollapse xAM images acquired at the same voltage steps were used as the blank value for data processing. For purified GV samples, B-mode images at the beginning of the pressure ramp and at the end of the postcollapse ramp were also acquired for concentration normalization. Unless otherwise noted, xAM imaging was performed at a focal depth of 5 mm by using the same imaging sequence for purified GV and cellular samples. Acoustic pressure was adjusted according to sample context: 350 kPa for UReKA1 or 489 kPa for UReKA2 was used for purified GV measurements to maximize phosphorylation-dependent dynamic range in buffer, whereas 419 kPa was used for cellular imaging of UReKA1 to improve the signal-to-background ratio in the more heterogeneous cellular environment while remaining below the acoustic (as distinct from hydrostatic) collapse threshold of the reporter; an acoustic pressure ramp (Figure S6b) confirmed that the nonlinear signal increased with pressure and plateaued without decline through 939 kPa, indicating that GVs were not collapsed at this imaging pressure.

### Statistical Analysis

For the mutational screen in Figure 1c, −PKA and + PKA conditions were compared separately for each mutant, and nominal unadjusted p-values were calculated using two-tailed paired *t* tests. Individual data points represent biological replicates, and the summary values are shown as mean ± s.d. For all *in vitro* experiments, the sample size is *N* = 3 biological replicates unless otherwise stated. For each biological replicate, there were technical replicates to account for variability in experimental procedures such as sample loading and pipetting. SEM was calculated by taking the values for the biological replicates, each of which was the mean of its technical replicates.

## Supplementary Material



## Data Availability

The mass spectrometry proteomics data have been deposited to the ProteomeXchange Consortium via the PRIDE partner repository with the data set identifier PXD071157 (https://www.ebi.ac.uk/pride/archive/projects/PXD071157). Plasmid maps and MATLAB scripts used for ultrasound acquisition and data processing are available from the corresponding author upon request.

## Notes

Including other characterization of UReKA; substrate-specificity analysis for UReKA; other UReKA variant screenings; ATP titration xAM response; phosphorylation kinetics; IRES optimization and acoustic pressure dependence; kinase-pathway specificity; cell viability; mammalian expression constructs (PDF)

## #

Koch Institute for Integrative Cancer Research, Massachusetts Institute of Technology, Cambridge, MA 02139, USA

## Abbreviations

UReKA: ultrasonic reporter of kinase activity
PKA: protein kinase A
GV: gas vesicle
GvpC: gas vesicle protein C
AKAR: A kinase activity reporter
xAM: cross-propagating amplitude modulation
LPP: lambda protein phosphatase
SDS-PAGE: sodium dodecyl sulfate–polyacrylamide gel electrophoresis
PKC: protein kinase C
CaMKII: calcium/calmodulin-dependent protein kinase II
FHA1: forkhead-associated domain 1
ATP: adenosine triphosphate
MS/MS: tandem mass spectrometry
BP: buoyancy purification
HEK293T: human embryonic kidney 293 cells expressing the SV40 Large T antigen
GvpA: gas vesicle protein A
IRES: internal ribosome entry site
Fsk: forskolin
IBMX: 3-isobutyl-1-methylxanthine
cAMP: cyclic adenosine monophosphate
SBR: signal-to-background ratio
