# Supplementary material for: Ultrasonic Reporter of Kinase Activity
Source: ACS Nano. 2026 Sep 9;20(37):25827–40. doi: 10.1021/acsnano.6c14484 (PMC13602928; doi:10.1021/acsnano.6c14484)
Supplement: Supplementary file 1 [file nn6c14484_si_001.pdf]

Ultrasonic Reporter of Kinase Activity

Jee Won Yang[1], *Zhiyang Jin*[2,†], *Ting-Yu Wang*[3], *Mikhail G. Shapiro*[1,4,5,*]

[1]Division of Chemistry and Chemical Engineering, California Institute of Technology; Pasadena, CA 91125, USA

[2]Division of Engineering and Applied Sciences, California Institute of Technology; Pasadena, CA 91125, USA

[3]Proteome Exploration Laboratory, California Institute of Technology; Pasadena, CA 91125, USA

[4]Andrew and Peggy Cherng Department of Medical Engineering, California Institute of Technology, Pasadena, California 91125, USA

[5]Howard Hughes Medical Institute, Pasadena, California 91125, USA

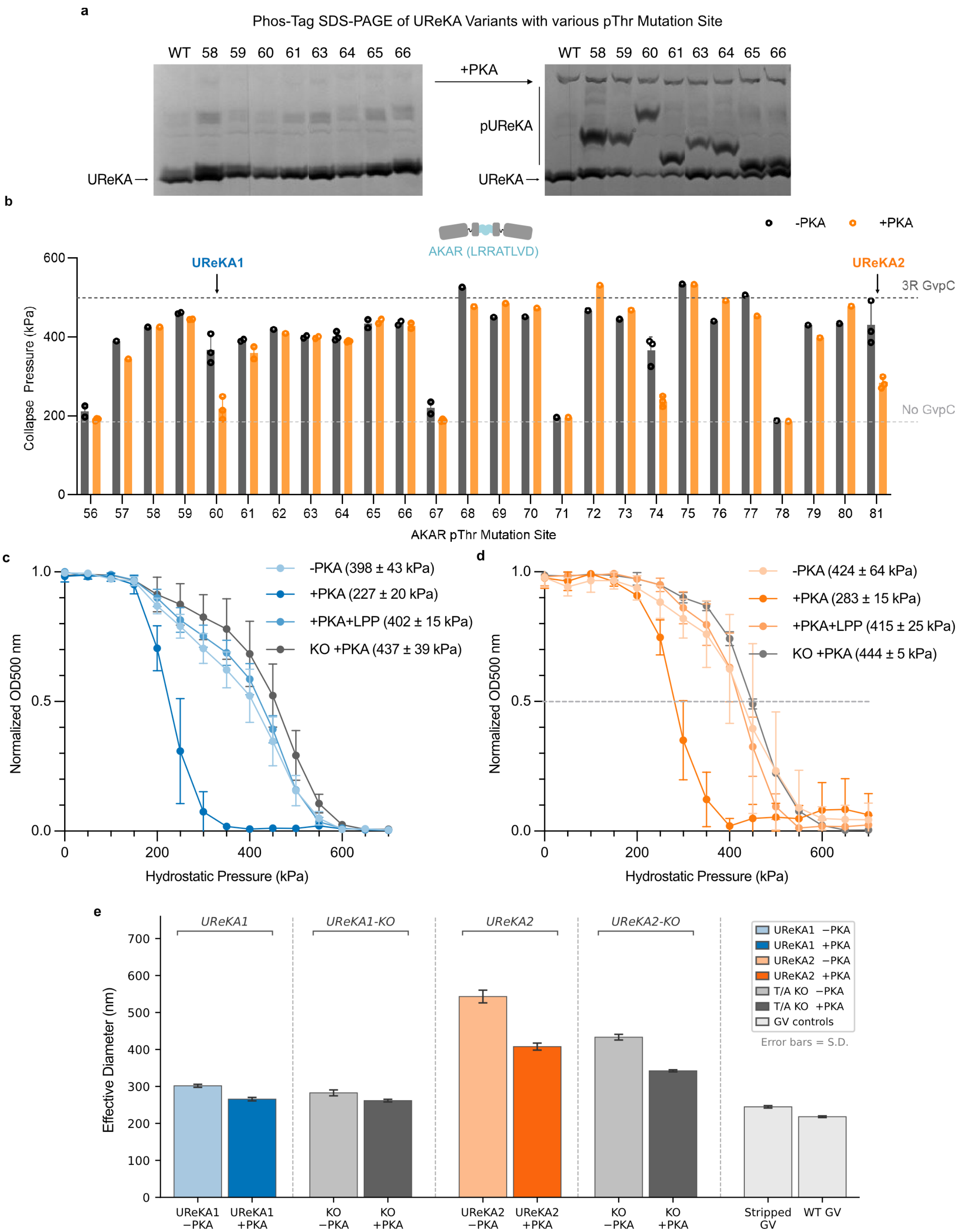
a
Phos-Tag SDS-PAGE of UReKA Variants with various pThr Mutation Site
WT 58 59 60 61 63 64 65 66
+PKA
WT 58 59 60 61 63 64 65 66
pUReKA
UReKA
UReKA
b
AKAR (LRRATLVD)
-PKA
+PKA
UReKA1
UReKA2
3R GvpC
No GvpC
Collapse Pressure (kPa)
AKAR pThr Mutation Site
c
-PKA (398 ± 43 kPa)
+PKA (227 ± 20 kPa)
+PKA+LPP (402 ± 15 kPa)
KO +PKA (437 ± 39 kPa)
Normalized OD500 nm
Hydrostatic Pressure (kPa)
d
-PKA (424 ± 64 kPa)
+PKA (283 ± 15 kPa)
+PKA+LPP (415 ± 25 kPa)
KO +PKA (444 ± 5 kPa)
Normalized OD500 nm
Hydrostatic Pressure (kPa)
e
UReKA1
UReKA1-KO
UReKA2
UReKA2-KO
UReKA1 −PKA
UReKA1 +PKA
UReKA2 −PKA
UReKA2 +PKA
T/A KO −PKA
T/A KO +PKA
GV controls
Error bars = S.D.
Effective Diameter (nm)
UReKA1 −PKA
UReKA1 +PKA
KO −PKA
KO +PKA
UReKA2 −PKA
UReKA2 +PKA
KO −PKA
KO +PKA
Stripped GV
WT GV

Figure S1. Other Characterization of UReKA. a) Coomassie Blue-stained Phos-Tag SDS-PAGE for various GvpC with AKAR sequence embedded in various mutation sites before (left) and after (right) incubation with PKA. b) Hydrostatic collapse pressure screening of UReKA variants with AKAR sequence at various phosphothreonine (pThr) position in GvpC; n=3 for 60, 74, 81, n=1 or 2 for others. Midpoint collapse pressure for each variant was determined from fitting a Boltzmann sigmoid function. Samples at OD_{500} 10 were incubated with 1mM ATP and with (orange) or without (black) 5,000 units of cAMP-activated PKA at 37˚C. Error bar indicates SEM based on curve fitting. Dashed lines indicate the mean signal of GVs with 3R GvpC (black) or without GvpC (gray). c) Normalized OD_{500} of UReKA1 at various conditions as a function of hydrostatic pressure without PKA, with PKA, with PKA and LPP, and T/A knockout (KO) used as a control; n=3. d) Normalized OD_{500} of UReKA2 at various conditions as a function of hydrostatic pressure without PKA, with PKA, with PKA and LPP, and T/A knockout (KO) used as a control; n=3. e) Effective diameter of UReKA1, UReKA2, and corresponding T/A KO variants measured by dynamic light scattering under -PKA and +PKA conditions with stripped GVs and WT GV as controls; n=6.

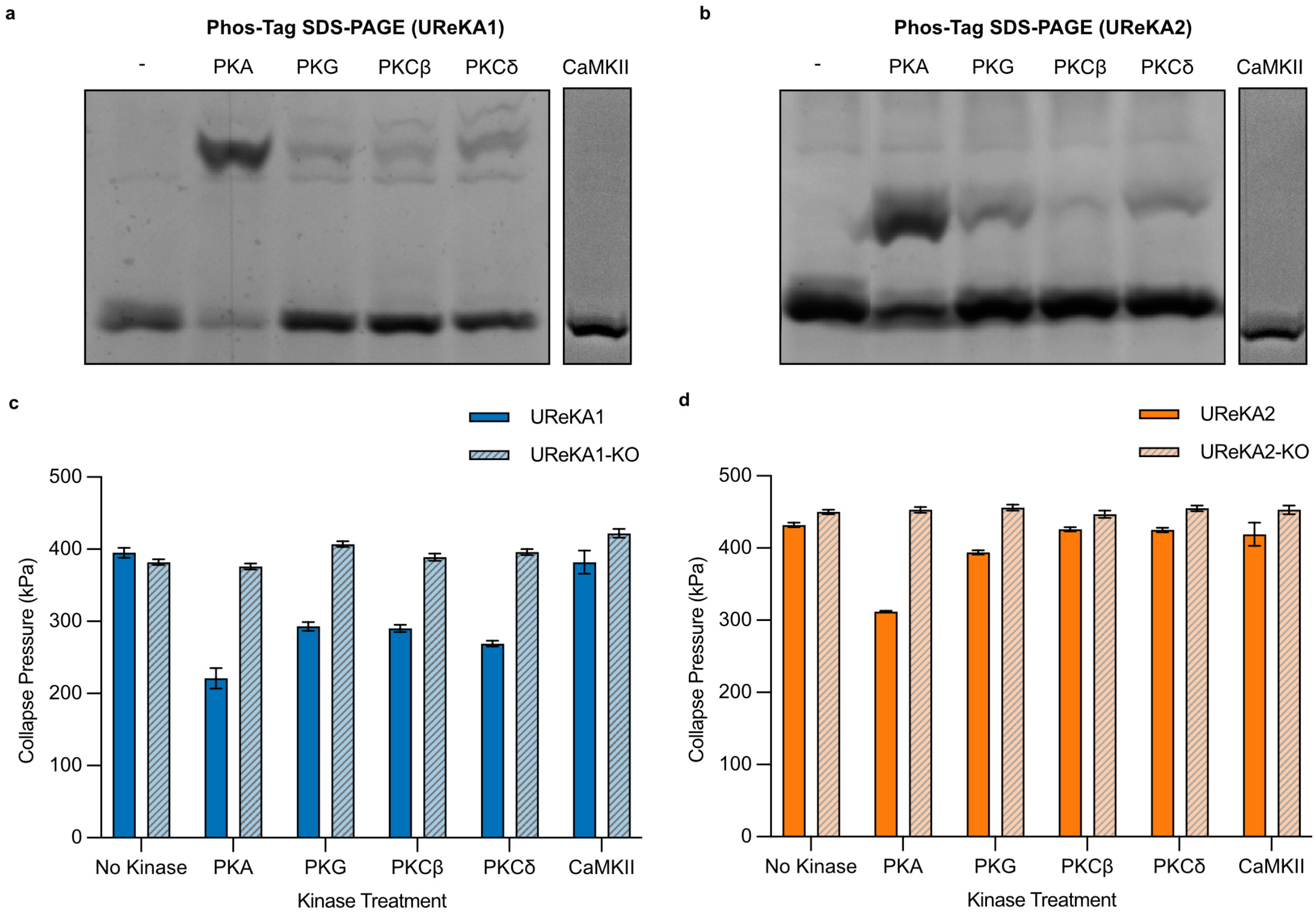

Figure S2. Substrate Specificity Analysis for UReKA. a) Coomassie Blue-stained Phos-Tag SDS-PAGE for UReKA1 incubated in the absence of kinase (-), or presence of PKA, PKG, PKCβ, PKCδ, or CaMKII. b) Phos-Tag SDS-PAGE for UReKA2 incubated in the absence of kinase (-), or presence of PKA, PKG, PKCβ, PKCδ, or CaMKII. c) Critical hydrostatic collapse pressure measured for UReKA1 and respective T/A KO (UReKA1-KO) incubated in the absence of kinase (-), or presence of PKA, PKG, PKCβ, PKCδ, or CaMKII. Error bars=s.d.; n=3. d) Critical hydrostatic collapse pressure measured for UReKA2 and respective T/A KO (UReKA2-KO) incubated in the absence of kinase (-), or presence of PKA, PKG, PKCβ, PKCδ, or CaMKII. Error bars=s.d.; n=3.

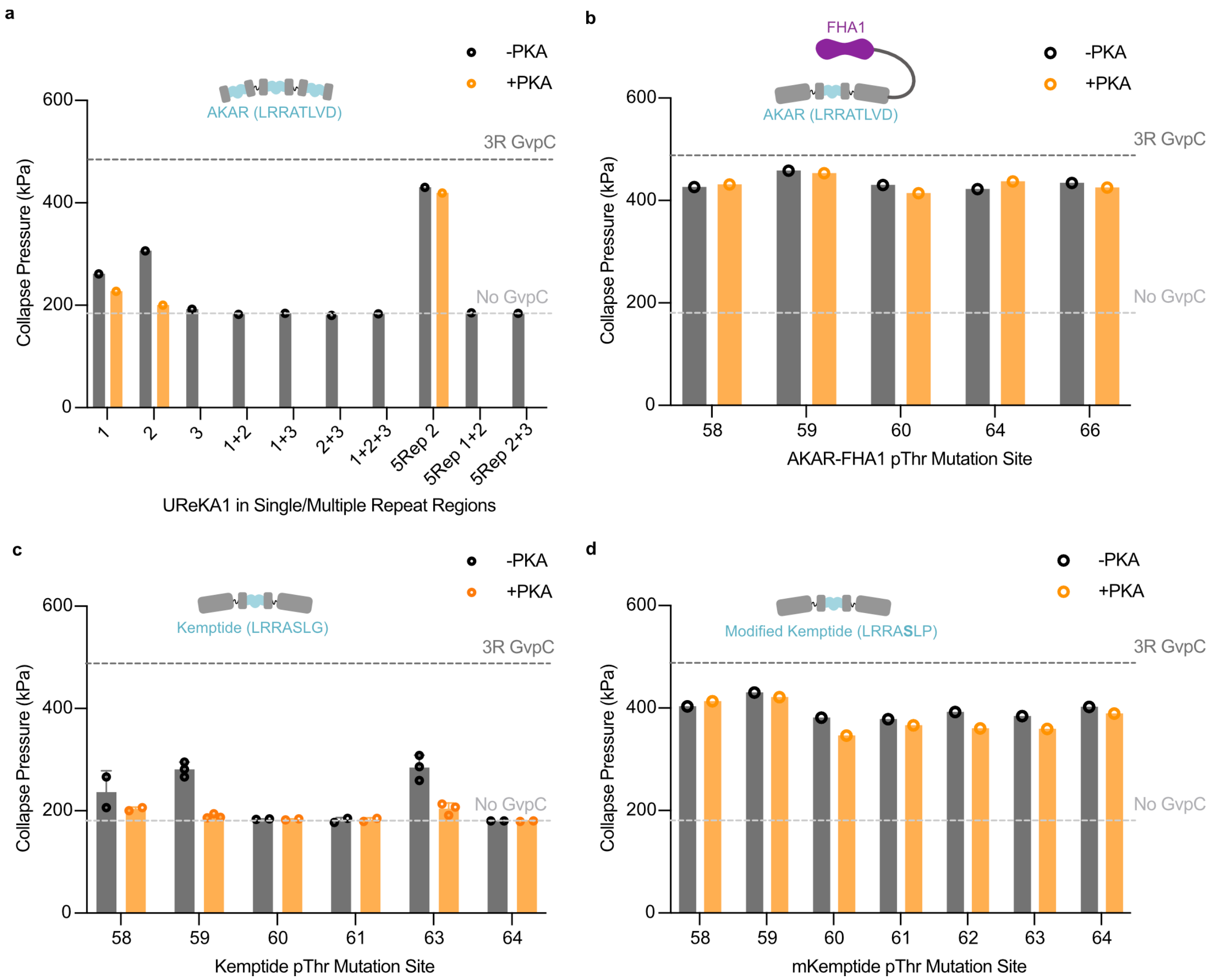

Figure S3. Other UReKA Variant Screenings. a)-d) Midpoint collapse pressure for each variant was determined from fitting a Boltzmann sigmoid function. Samples at OD_{500} 10 were incubated with 1 mM ATP and with (orange) or without (black) 5,000 units of cAMP-activated PKA at 37 °C. Error bar indicates SEM based on curve fitting. Dashed lines indicate the mean signal of GVs with 3R GvpC (black) or without GvpC (gray). a) Hydrostatic collapse pressure screening of AKAR sequence in various repeats of GvpC (1=27, 2=60, 3=93; 5Rep=5-repeat GvpC); n=1. b) Hydrostatic collapse pressure screening of AKAR-FHA1 UReKA variants; n=1. c) Hydrostatic collapse pressure screening of Kemptide-UReKA variants; n=3 for 59 and 63,

n=2 for others. d) Hydrostatic collapse pressure screening of modified Kemptide-UReKA variants; n=1.

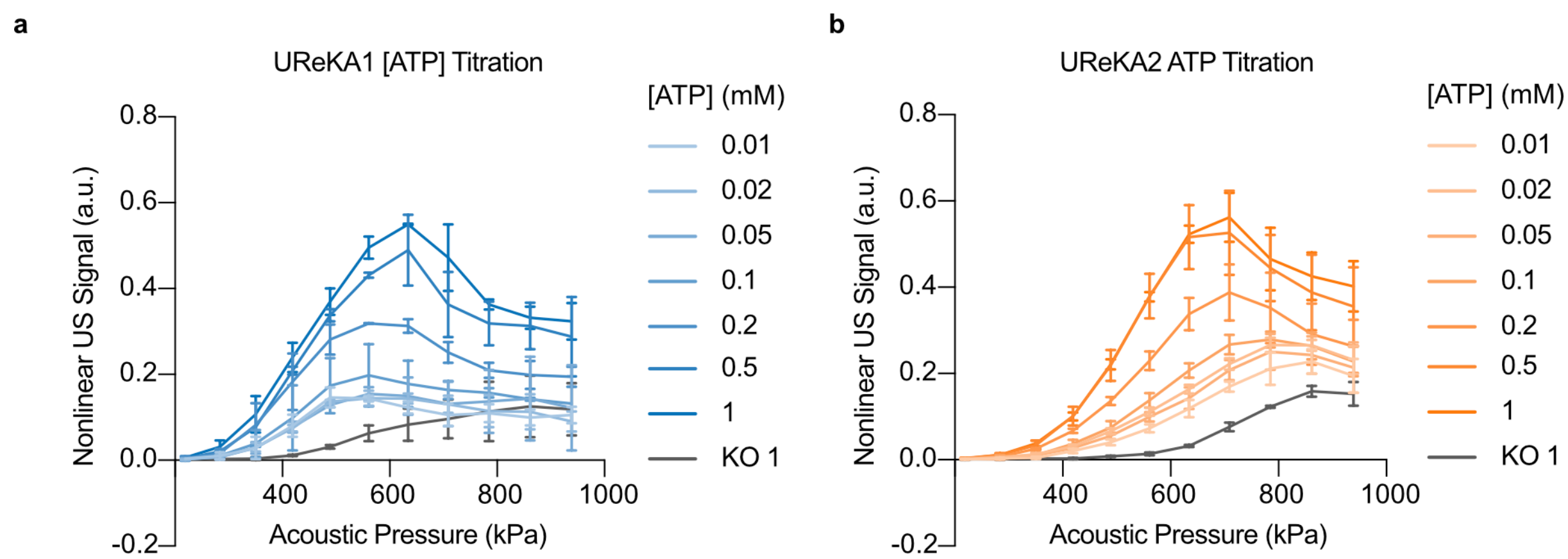

Figure S4. ATP titration xAM response. a) Nonlinear ultrasound signal of UReKA1 (blue) incubated with PKA and various concentrations of ATP from 10μM to 1mM with respective T/A KO control (gray); n=3. b) Nonlinear ultrasound signal of UReKA2 (orange) incubated with PKA and various concentrations of ATP from 10μM to 1mM with respective T/A KO control (gray); n=3.

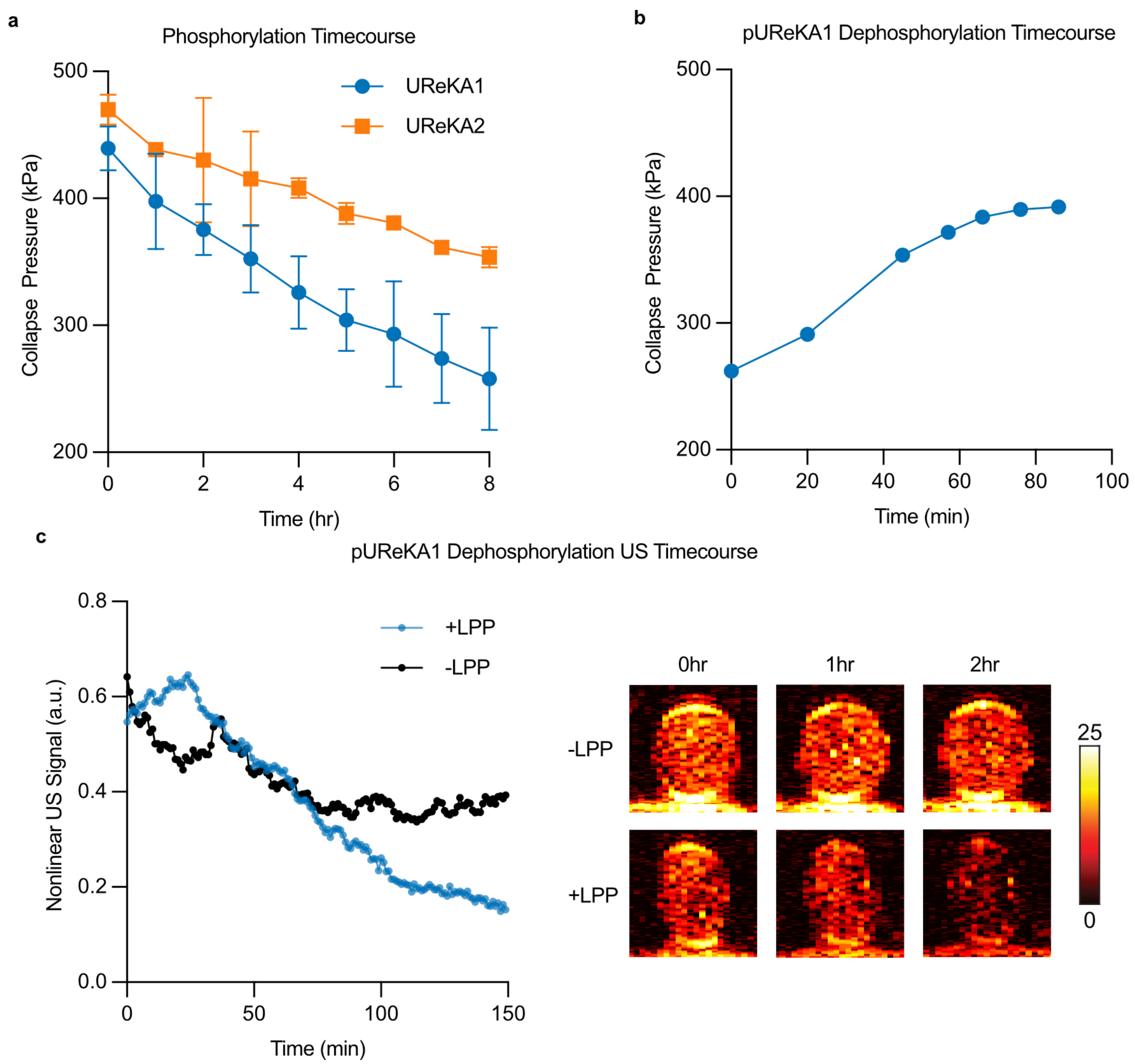

Figure S5. Time-resolved Analysis of UReKA Mechanism. a) Critical collapse pressure of UReKA1 and UReKA2 measured over time during phosphorylation. Error bars indicate S.D; n=4. b) Recovery of collapse pressure for phosphorylated UReKA1 (pUReKA1) following dephosphorylation with LPP over time; n=1. c) (left) Time-dependent nonlinear ultrasound signal from pUReKA1 in the presence (+LPP) or absence (-LPP) of LPP; n=1. (right) Representative ultrasound images of pUReKA1 at 0, 1, and 2 hours under -LPP and +LPP conditions.

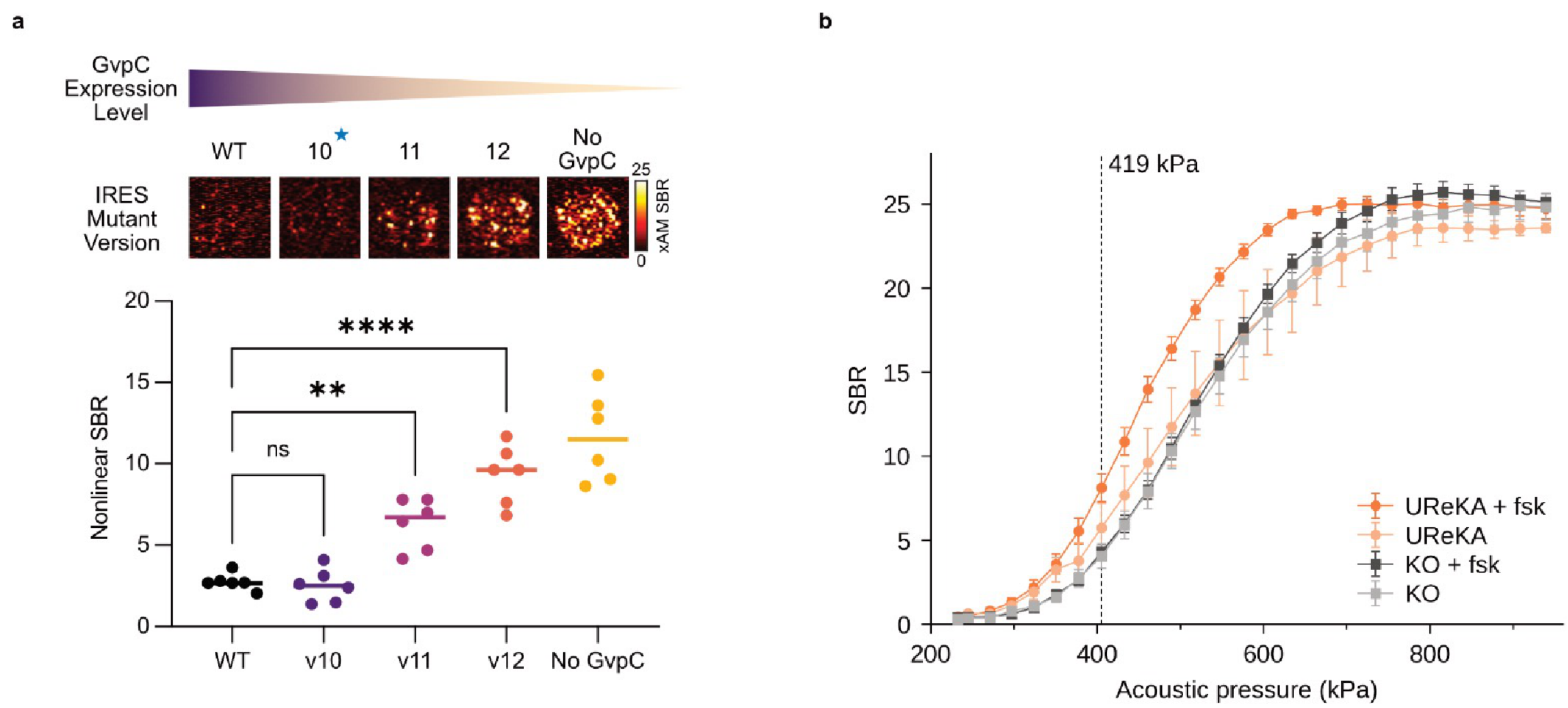

Figure S6. IRES optimization and acoustic pressure dependence of cellular UReKA. a) Representative nonlinear xAM image at 419 kPa (top) of various UReKA GVs expressed with mutant IRES from v10-12 upstream of GvpC with respective SBR (below); n=6. Asterisk indicate UReKA1-IRESv10 variant used for downstream investigations. b) Nonlinear (xAM) ultrasound signal-to-background ratio (SBR) of the UReKA1-IRESv10 reporter (UReKA) and its phosphorylation-null threonine-to-alanine control (KO), each incubated with (+ fsk) or without forskolin, plotted as a function of applied acoustic pressure over a ramp from 219 to 939 kPa. The vertical dashed line marks the 419 kPa acoustic pressure used to acquire the cellular xAM images. Error bars indicate S.D.; n = 6.

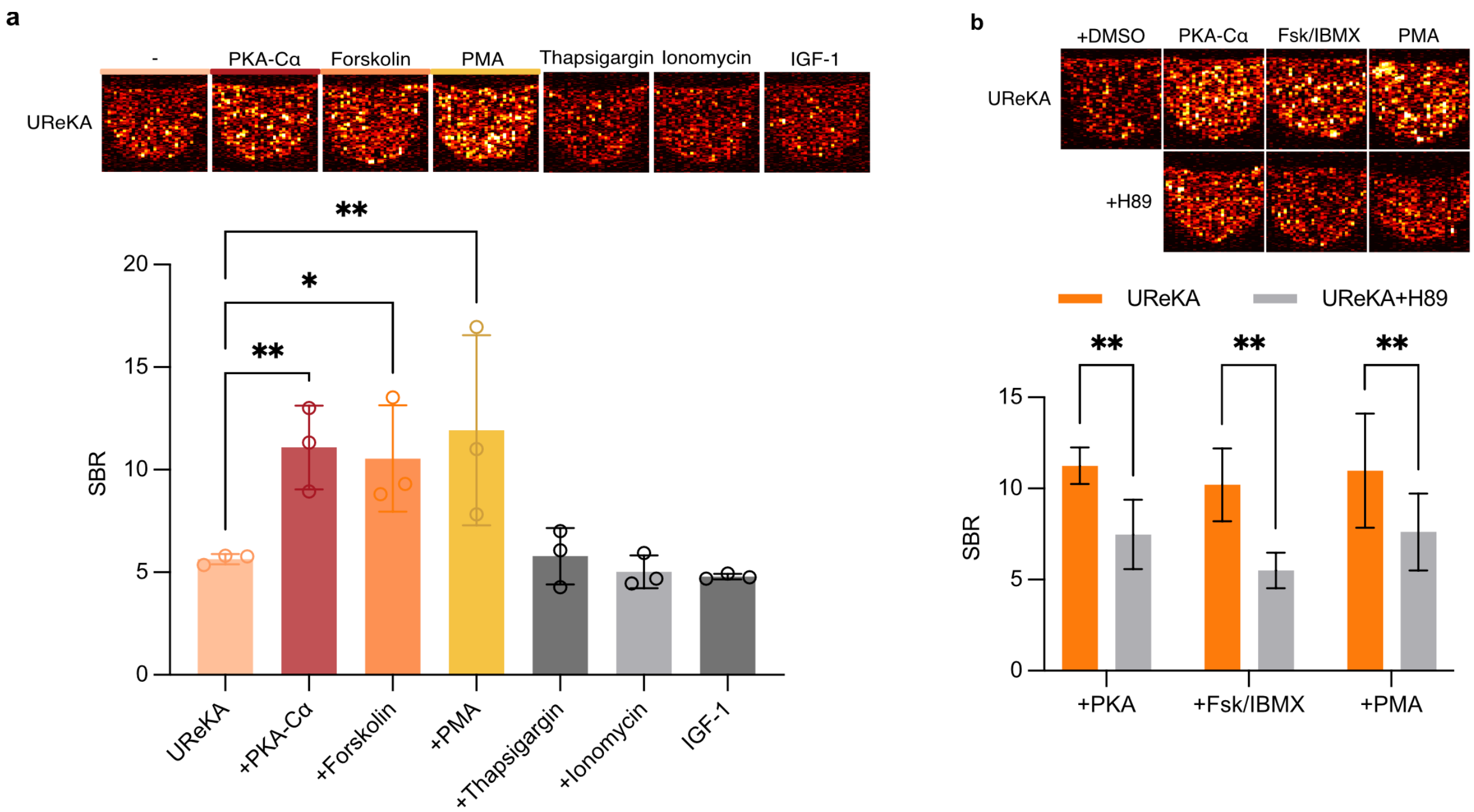

Figure S7. Specificity of UReKA Activation by Various Kinase signaling pathways. Bars represent mean ± s.d. Statistical significance is indicated (*p<0.05, **p <0.01). a) Representative nonlinear ultrasound images (top) and quantification of SBR (bottom) for UReKA-expressing samples with various kinase pathway activators, including co-expression of PKA-Cα, treatment with forskolin, PMA, thapsigargin, ionomycin, and IGF-1; n=3. b) Representative nonlinear ultrasound images (top) and corresponding SBR (bottom) for UReKA samples treated with PKA-Cα, forskolin, or PMA in the presence or absence of the PKA inhibitor H89; n=3.

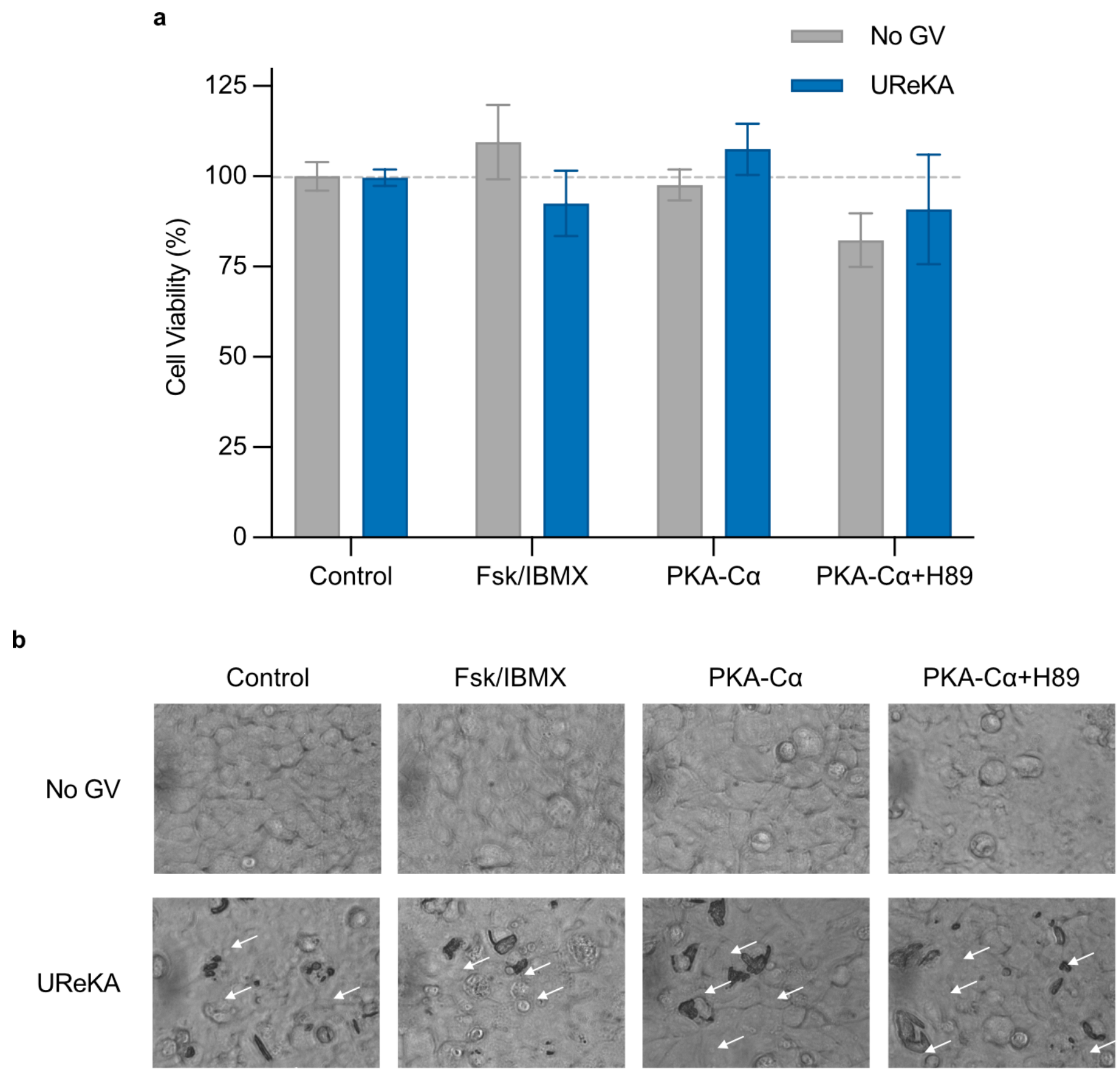

Figure S8. Cell Viability Assessment by CCK-8 Assay. a) Cell viability of HEK293T cells without GVs (No GV, gray) and expressing UReKA1 (UReKA, blue) under the indicated pharmacological conditions, measured by CCK-8 assay at 72h post-transfection. Viability is expressed as a percentage relative to the unstimulated No GV control. Conditions: vehicle control (−), Fsk/IBMX stimulation, PKA-Cα overexpression (PKA-Cα), and PKA-Cα overexpression with H89 inhibitor (PKA-Cα + H89). Bars represent mean ± s.e.m.; n=3. Dashed line indicates 100% viability. b) Phase contrast images of HEK293T cells with or without UReKA transfection under indicated treatment conditions at 72 hr post-transfection. In UReKA-transfected cells, GV clusters appear as dark refractile bodies, highlighted by white arrows.

Table S1. Mammalian expression constructs used in this study.

Construct	Backbone	Size (bp)	Key elements
gvpA-IRESv10-gvpC (UReKA1)	pCMV-Sport	7,518	CMV enhancer/promoter · gvpA · IRESv10 (mutant IRES2) · UReKA1 gvpC (AKAR motif in repeat 2) · P2A · emiRFP670 · WPRE · hGH polyA · AmpR · ColE1 ori
gvpNJKFGWV assembly-factor	pCMV-Sport	10,484	CMV enhancer/promoter · P2A-linked gvpN-J-K-F-G-W-V · EmGFP · WPRE · hGH polyA · AmpR · ColE1 ori

Total plasmid sizes and principal functional elements of the two mammalian expression constructs. Using an average double-stranded DNA mass of ~650 g/mol per bp, the transfected quantities of 280 fmol and 70 fmol correspond to approximately 1.37 μg and 0.48 μg, respectively—a 4:1 molar ratio favoring the gvpA-containing cassette—with pUC19 supplemented to a 2 μg total. Annotated plasmid maps (SnapGene/GenBank) are available upon request.
